# Supplementary material for: Simulating the quantum switch with quantum circuits is computationally hard
Source: Nat Commun. 2025 Nov 20;16:10216. doi: 10.1038/s41467-025-64996-6 (PMC12635086; doi:10.1038/s41467-025-64996-6)
Supplement: Supplementary file 1 — Supplementary Information [file 41467_2025_64996_MOESM1_ESM.pdf]

Supplementary Information for: Simulating the quantum switch with quantum circuits is computationally hard

Jessica Bavaresco,[1,2] Hlér Kristjánsson,[3,4,5,6] Mio Murao,[5,7] Tatsuki Odake,[5]
Marco Túlio Quintino,[2] Philip Taranto,[8,5] and Satoshi Yoshida[5]

[1] *Department of Applied Physics, University of Geneva, 1205 Geneva, Switzerland*
[2] *Sorbonne Université, CNRS, LIP6, F-75005 Paris, France*
[3] *Perimeter Institute for Theoretical Physics, 31 Caroline Street North, Waterloo, Ontario, N2L 2Y5, Canada*
[4] *Institute for Quantum Computing, University of Waterloo, 200 University Avenue West, Waterloo, Ontario, N2L 3G1, Canada*
[5] *Department of Physics, Graduate School of Science, The University of Tokyo, Hongo 7-3-1, Bunkyo-ku, Tokyo 113-0033, Japan*
[6] *Department of Computer Science and Operations Research, Université de Montréal, Montréal, Québec, H3T 1J4, Canada*
[7] *Trans-scale Quantum Science Institute, The University of Tokyo, Hongo 7-3-1, Bunkyo-ku, Tokyo 113-0033, Japan*
[8] *Department of Physics & Astronomy, University of Manchester, Manchester M13 9PL, United Kingdom*

Contents

1. Supplementary Note 1 – Definitions and Choi operators 1
   1.1. The quantum switch Choi operator and Choi operator fidelity 2
   1.2. Quantum combs, probabilistic quantum combs, and projectors 2
   1.3. QC-CCs and probabilistic QC-CCs 3

2. Supplementary Note 2 – General simulation scenarios 4
   2.1. The action of the quantum switch on bipartite quantum channels 5
   2.2. The action of the quantum switch on quantum instruments 6
   2.3. The relationship with Stinespring dilation of quantum channels 6
   2.4. Theorem 1: A simulation for bipartite unitary channels U_A and bipartite general channels B 8

3. Supplementary Note 3 – Full proof of Theorem 2 11
   3.1. Lemma 1 12
   3.2. Lemma 2 (for $M, N \in \mathbb{N}^+$) 13
   3.3. Lemma 3 (for $M, N \in \mathbb{N}^+$) 14
   3.4. Lemma 4 (for $M < 4^n/2 + 2$ and $N = 1$) 15
   3.5. Lemma 5 (for $\max\{M, N\} \leq \max\{2, d-1\}$) 22
   3.6. Lemma 6 25
   3.7. Lemma 7 26

4. Supplementary Note 4 – Possible restricted simulations for qubit channels 27

5. Supplementary Note 5 – No-go results for unitary channels 29

6. Supplementary Note 6 – Efficient certification that a matrix is positive semidefinite 29

References 30

Supplementary Note 1 – Definitions and Choi operators

In this section, we explicitly define the key higher-order transformations used in the main text in terms of their Choi operators, which are crucial for the SDP implementation of our methods.

1.1 The quantum switch Choi operator and Choi operator fidelity

We begin with the Choi operator of the quantum switch transformation [1]. The quantum switch higher-order transformation $\mathcal{S}$ defined in the main text in terms of its Kraus decomposition [Eqs. (1) and (2)], can be equivalently expressed by its associated Choi operator $S \in \mathcal{L}(\mathcal{H}^{c_I} \otimes \mathcal{H}^{t_I} \otimes \mathcal{H}^{A_I} \otimes \mathcal{H}^{A_O} \otimes \mathcal{H}^{B_I} \otimes \mathcal{H}^{B_O} \otimes \mathcal{H}^{t_O} \otimes \mathcal{H}^{c_O})$. Let the vector $|S\rangle\rangle$ be defined as

$$|S\rangle\rangle := |0\rangle^{c_I} |\mathbb{1}\rangle\rangle^{t_I A_I} |\mathbb{1}\rangle\rangle^{A_O B_I} |\mathbb{1}\rangle\rangle^{B_O t_O} |0\rangle^{c_O} + |1\rangle^{c_I} |\mathbb{1}\rangle\rangle^{t_I B_I} |\mathbb{1}\rangle\rangle^{B_O A_I} |\mathbb{1}\rangle\rangle^{A_O t_O} |1\rangle^{c_O}, \tag{1}$$

where $|\mathbb{1}\rangle\rangle^{XY} := \sum_i |i\rangle^X |i\rangle^Y$ is the vector related to the Choi operator $|\mathbb{1}\rangle\rangle\langle\langle\mathbb{1}|$ of an identity channel from space X to Y. Then, the Choi operator of the quantum switch is defined as

$$S := |S\rangle\rangle\langle\langle S|. \tag{2}$$

This is the operator S that appears in the first constraint of the SDPs associated to the primal and dual problem, presented in the main text [Eqs. (16) and (17)].

The Choi operator of the quantum channel $\mathcal{S}(A, B)$ that results from the action of the quantum switch transformation $\mathcal{S}$ on input channels A and B can be expressed in terms of their respective Choi operators S, J^A, and J^B, as $S * (J^A \otimes J^B) \in \mathcal{L}(\mathcal{H}^{c_I} \otimes \mathcal{H}^{t_I} \otimes \mathcal{H}^{t_O} \otimes \mathcal{H}^{c_O})$, where $*$ is the link product defined in the Methods section of the main text [Eq. (15)].

In the case considered in the main text where the quantum switch has a fixed input state for its control and target systems, respectively given by $\sigma_c = |+\rangle\langle+| \in \mathcal{L}(\mathcal{H}^{c_I})$ and $\rho_t = |0\rangle\langle 0| \in \mathcal{L}(\mathcal{H}^{t_I})$, we defined the resulting transformation as $\mathcal{S}_{+0}(\cdot,\cdot) := \mathcal{S}(\cdot,\cdot)[|+\rangle\langle+| \otimes |0\rangle\langle 0|]$. This higher-order operation has an associated Choi operator $S_{+0} \in \mathcal{L}(\mathcal{H}^{A_I} \otimes \mathcal{H}^{A_O} \otimes \mathcal{H}^{B_I} \otimes \mathcal{H}^{B_O} \otimes \mathcal{H}^{t_O} \otimes \mathcal{H}^{c_O})$ given by

$$S_{+0} := \Big(|+\rangle\langle+| \otimes |0\rangle\langle 0| \Big) * S \tag{3}$$

$$= |S_{+0}\rangle\rangle\langle\langle S_{+0}|, \tag{4}$$

where

$$|S_{+0}\rangle\rangle := \Big(\langle+|^{c_I} \langle 0|^{t_I} \otimes \mathbb{1} \Big) |S\rangle\rangle = \frac{1}{\sqrt{2}} \Big(|0\rangle^{A_I} |\mathbb{1}\rangle\rangle^{A_O B_I} |\mathbb{1}\rangle\rangle^{B_O t_O} |0\rangle^{c_O} + |1\rangle^{B_I} |\mathbb{1}\rangle\rangle^{B_O A_I} |\mathbb{1}\rangle\rangle^{A_O t_O} |1\rangle^{c_O} \Big). \tag{5}$$

We now define the Choi operator fidelity. Let $\widetilde{\mathcal{S}}$ be a higher-order transformation that acts on the same spaces as the quantum switch $\mathcal{S}$, and let $\widetilde{\mathcal{S}}_{+0} := \widetilde{\mathcal{S}}(\cdot,\cdot)[|+\rangle\langle+| \otimes |0\rangle\langle 0|]$ be $\widetilde{\mathcal{S}}$ with fixed input states for its control and target systems. The Choi operator fidelity between $\mathcal{S}_{+0}$ and any higher-order operation $\widetilde{\mathcal{S}}_{+0}$, with Choi operator $\widetilde{S}_{+0}$ is given by

$$F(\mathcal{S}_{+0}, \widetilde{\mathcal{S}}_{+0}) := \frac{1}{(d_{A_O} d_{B_O})^2} \mathrm{tr}(S_{+0}\, \widetilde{S}_{+0}) = \frac{1}{(d_{A_O} d_{B_O})^2} \Big| \langle s_{+0}| \, \widetilde{S}_{+0} \, |s_{+0}\rangle \Big|, \tag{6}$$

where the factor $d_{A_O} d_{B_O} = \mathrm{tr}(S_{+0}) = \mathrm{tr}(\widetilde{S}_{+0})$ ensures that $F(\mathcal{S}_{+0}, \widetilde{\mathcal{S}}_{+0}) \in [0, 1]$.

1.2 Quantum combs, probabilistic quantum combs, and projectors

A probabilistic quantum comb is a quantum comb that yields a classical output with a certain probability. In our case, we consider probabilistic quantum combs that have two possible classical outcomes, success or failure. We recall that such transformations can be implemented by a quantum comb that additionally output a flag system that encodes the success or failure outcome, followed by a dichotomic quantum measurement of the flag system. In the case of a quantum comb $\mathcal{C} = \mathcal{C}_s + \mathcal{C}_f$ that performs a probabilistic simulation of the quantum switch, its associated Choi operator is given by $C = C_s + C_f$, where $C, C_s, C_f \in \mathcal{L}(\mathcal{H}^{c_I} \otimes \mathcal{H}^{t_I} \otimes (\mathcal{H}^{A_I} \otimes \mathcal{H}^{A_O})^{\otimes k_A} \otimes (\mathcal{H}^{B_I} \otimes \mathcal{H}^{B_O})^{\otimes k_B} \otimes \mathcal{H}^{t_O} \otimes \mathcal{H}^{c_O})$.

These operators are characterised by

$$C_s \geq 0 \tag{7}$$

$$C_f = C - C_s \geq 0 \tag{8}$$

$$\mathrm{tr}(C) = d_{c_I} d_{t_I} d_{A_O}^{k_A} d_{B_O}^{k_B} \tag{9}$$

$$C = \mathbb{P}_k(C), \tag{10}$$

where $k = k_A + k_B$ is the total number of slots in the quantum comb C, and $\mathbb{P}_k$ is the projector onto the subspace spanned by k-slot quantum combs.

The projector $\mathbb{P}_k$ is defined in full generality in Ref. [2]. For sake of completeness, we explicitly write $\mathbb{P}_k$ here in the cases where $k \in \{2,3,4\}$, which are the ones involved in our numerical calculations. To simplify the notation in the definition of the projector, let us define a k-slot quantum comb by its Choi operator $C \in \mathcal{L}(\mathcal{H}^P \otimes \mathcal{H}^{I_1} \otimes \mathcal{H}^{O_1} \otimes \ldots \otimes \mathcal{H}^{I_k} \otimes \mathcal{H}^{O_k} \otimes \mathcal{H}^F)$. Then, let us define the trace-and-replace operation acting on the subspace $\mathcal{H}^X$ of an operator C as

$$ {}_X C \coloneqq \mathrm{tr}_X(C) \otimes \frac{\mathbb{1}^C}{d_C}. \tag{11}$$

We are now ready to explicitly write the projector $\mathbb{P}_k$ for $k \in \{2,3,4\}$. For the case where $k = 2$,

$$\mathbb{P}_2(C) = C - {}_F C + {}_{O_2F} C - {}_{I_2O_2F} C + {}_{O_1I_2O_2F} C - {}_{I_1O_1I_2O_2F} C + {}_{PI_1O_1I_2O_2F} C. \tag{12}$$

For the case where $k = 3$,

$$\begin{aligned}\mathbb{P}_3(C) = C &- {}_F C + {}_{O_3F} C - {}_{I_3O_3F} C + {}_{O_2I_3O_3F} C - {}_{I_2O_2I_3O_3F} C + {}_{O_1I_2O_2I_3O_3F} C \\ &- {}_{I_1O_1I_2O_2I_3O_3F} C + {}_{PI_1O_1I_2O_2I_3O_3F} C.\end{aligned} \tag{13}$$

Finally, for the case where $k = 4$,

$$\begin{aligned}\mathbb{P}_4(C) = C &- {}_F C + {}_{O_4F} C - {}_{I_4O_4F} C + {}_{O_3I_4O_4F} C - {}_{I_3O_3I_4O_4F} C + {}_{O_2I_3O_3I_4O_4F} C - {}_{I_2O_2I_3O_3I_4O_4F} C \\ &+ {}_{O_1I_2O_2I_3O_3I_4O_4F} C - {}_{I_1O_1I_2O_2I_3O_3I_4O_4F} C + {}_{PI_1O_1I_2O_2I_3O_3I_4O_4F} C.\end{aligned} \tag{14}$$

For convenience, we also define the dual affine projector used in the dual formulation of the SDP in the main text [Eq. (17)]. Given a set of operators $\mathcal{A}$, its dual affine set $\mathcal{B}$ is the set of all operators B such that $\mathrm{tr}(A^\dagger B) = 1$ for all $A \in \mathcal{A}$. Hence, the dual affine set of the set of Choi operators of quantum combs $C \in \mathcal{L}(\mathcal{H}^P \otimes \mathcal{H}^{I_1} \otimes \mathcal{H}^{O_1} \otimes \ldots \otimes \mathcal{H}^{I_k} \otimes \mathcal{H}^{O_k} \otimes \mathcal{H}^F)$ is the set of all operators $\Gamma \in \mathcal{L}(\mathcal{H}^P \otimes \mathcal{H}^{I_1} \otimes \mathcal{H}^{O_1} \otimes \ldots \otimes \mathcal{H}^{I_k} \otimes \mathcal{H}^{O_k} \otimes \mathcal{H}^F)$ such that $\mathrm{tr}(C\Gamma) = 1$. The operators in the dual affine set of quantum combs are themselves a particular case of quantum combs, and can be characterized by projectors $\overline{\mathbb{P}}_k$ given by [2]

$$\overline{\mathbb{P}}_k(\Gamma) = \Gamma - \mathbb{P}_k(\Gamma) + {}_{PI_1I_2\ldots I_kO_kF}\Gamma. \tag{15}$$

In our numerical calculations, we used the code available in the repository of Ref. [2] to generate the above projector constraints.

1.3 QC-CCs and probabilistic QC-CCs

We now present the explicit constraints for QC-CC transformations. QC-CCs and probabilistic QC-CCs have been defined in full generality and for any number of slots in Ref. [3]. Here, we write these definitions explicitly for the cases of $k = 2$ and $k = 3$ slots, which were used in our numerical calculations. Since we only evaluated the maximal probability of simulating the quantum switch with a QC-CC in the restricted simulation scenario, where the input control and target systems are fixed, we write the explicit constraints for a probabilistic QC-CC with fixed input systems (compared to the definition of quantum combs in this section, this is the equivalent of a scenario where $d_P = 1$).

Unlike the case of quantum combs, to define the Choi operator $W = W_s + W_f$ associated to a probabilistic QC-CC, where $W, W_s, W_f \in \mathcal{L}(\mathcal{H}^{I_1} \otimes \mathcal{H}^{O_1} \otimes \ldots \otimes \mathcal{H}^{I_k} \otimes \mathcal{H}^{O_k} \otimes \mathcal{H}^{F})$, it is necessary but not sufficient to say that $W_s \geq 0$, $W_f = W - W_s \geq 0$ and W is a QC-CC, as individual constraints must be applied to W_s and W_f as well to ensure validity of the overall transformation.

In the case where $k = 2$, we have that

$$W = W_s + W_f \tag{16}$$
$$\operatorname{tr}(W) = d_{O_1} d_{O_2}, \tag{17}$$

where

$$W_s = W_s^{12F} + W_s^{21F}, \qquad W_f = W_f^{12F} + W_f^{21F}, \tag{18}$$

such that $W_s^{12F} \geq 0$, $W_s^{21F} \geq 0$, $W_f^{12F} \geq 0$, and $W_f^{21F} \geq 0$, and additionally $W^{12F} := W_s^{12F} + W_f^{12F}$ must be a quantum comb with the order $I_1 O_1 I_2 O_2 F$, and $W^{21F} := W_s^{21F} + W_f^{21F}$ must be a quantum comb with the order $I_2 O_2 I_1 O_1 F$.

In the case where $k = 3$, we have that

$$W = W_s + W_f \tag{19}$$
$$\operatorname{tr}(W) = d_{O_1} d_{O_2} d_{O_3}. \tag{20}$$

where

$$W_s = W_s^{123F} + W_s^{132F} + W_s^{213F} + W_s^{231F} + W_s^{312F} + W_s^{321F} \tag{21}$$
$$W_f = W_f^{123F} + W_f^{132F} + W_f^{213F} + W_f^{231F} + W_f^{312F} + W_f^{321F}, \tag{22}$$

where $W_s^{xyzF} \geq 0$ and $W_f^{xyzF} \geq 0$ for all $xyz \in \text{Perm}(1,2,3)$. Moreover, we define $W^{xyzF} := W_s^{xyzF} + W_f^{xyzF}$ for all $xyz \in \text{Perm}(1,2,3)$ and impose that the operators W^{123F} and W^{132F} must satisfy

$${}_F W^{123F} = {}_{O_3 F} W^{123F}, \qquad {}_F W^{132F} = {}_{O_2 F} W^{132F} \tag{23}$$
$${}_{I_3 O_3 F} W^{123F} = {}_{O_2 I_3 O_3 F} W^{123F}, \qquad {}_{I_2 O_2 F} W^{132F} = {}_{O_3 I_2 O_2 F} W^{132F} \tag{24}$$
$${}_{I_2 O_2 I_3 O_3 F} W^{123F} + {}_{I_3 O_3 I_2 O_2 F} W^{132F} = {}_{O_1}({}_{I_2 O_2 I_3 O_3 F} W^{123F} + {}_{I_3 O_3 I_2 O_2 F} W^{132F}). \tag{25}$$

Similarly, W^{213F} and W^{231F} must satisfy

$${}_F W^{213F} = {}_{O_3 F} W^{213F}, \qquad {}_F W^{231F} = {}_{O_1 F} W^{231F} \tag{26}$$
$${}_{I_3 O_3 F} W^{213F} = {}_{O_1 I_3 O_3 F} W^{213F}, \qquad {}_{I_1 O_1 F} W^{231F} = {}_{O_3 I_1 O_1 F} W^{231F} \tag{27}$$
$${}_{I_1 O_1 I_3 O_3 F} W^{213F} + {}_{I_3 O_3 I_1 O_1 F} W^{231F} = {}_{O_2}({}_{I_1 O_1 I_3 O_3 F} W^{213F} + {}_{I_3 O_3 I_1 O_1 F} W^{231F}). \tag{28}$$

Finally, W^{312F} and W^{321F} must satisfy

$${}_F W^{312F} = {}_{O_2 F} W^{312F}, \qquad {}_F W^{321F} = {}_{O_1 F} W^{321F} \tag{29}$$
$${}_{I_2 O_2 F} W^{312F} = {}_{O_1 I_2 O_2 F} W^{312F}, \qquad {}_{I_1 O_1 F} W^{321F} = {}_{O_2 I_1 O_1 F} W^{321F} \tag{30}$$
$${}_{I_1 O_1 I_2 O_2 F} W^{312F} + {}_{I_2 O_2 I_1 O_1 F} W^{321F} = {}_{O_3}({}_{I_1 O_1 I_2 O_2 F} W^{312F} + {}_{I_2 O_2 I_1 O_1 F} W^{321F}). \tag{31}$$

Supplementary Note 2 – General simulation scenarios

In this section, we discuss the differences between simulation scenarios where the quantum switch acts on general quantum channels, on only part of general quantum channels, or on quantum instruments, highlighting how they relate to the action of the quantum switch on unitary channels. We also demonstrate how the switch simulation that we introduced in the circuit related to Thm. 1 [written in the main text and reproduced in here as Eq. (36)] fits into

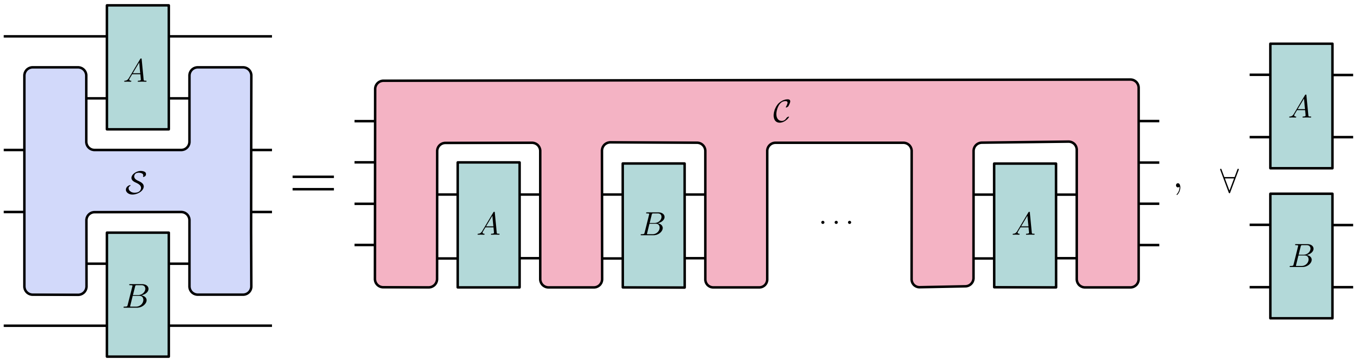

Supplementary Figure 1. **General simulation of the quantum switch.** A higher-order transformation $\mathcal{C}$, which can be a quantum comb or a QC-CC, that acts on part of several copies of input bipartite quantum channels A and B is a simulation of the quantum switch $\mathcal{S}$ if it reproduces the action of the quantum switch on all arbitrary pairs of bipartite channels A and B.

this context.

2.1 The action of the quantum switch on bipartite quantum channels

The first simulation scenario discussed in the main text is the one where the quantum switch $\mathcal{S}$ acts on the "entire" quantum channels A and B, as represented in Fig. 2, in the main text. Formally, this is the case where the quantum switch $\mathcal{S}$ acts on a pair of single-party (single input system, single output system) channels $A : \mathcal{L}(\mathcal{H}^{A_I}) \to \mathcal{L}(\mathcal{H}^{A_O})$ and $B : \mathcal{L}(\mathcal{H}^{B_I}) \to \mathcal{L}(\mathcal{H}^{B_O})$, and outputs another channel $\mathcal{S}(A, B) : \mathcal{L}(\mathcal{H}^{c_I} \otimes \mathcal{H}^{t_I}) \to \mathcal{L}(\mathcal{H}^{c_O} \otimes \mathcal{H}^{t_O})$. However, quantum theory also allows one to apply the quantum switch on *parts* of bipartite (two input systems, two output systems) channels $A : \mathcal{L}(\mathcal{H}^{A_I} \otimes \mathcal{H}^{A'_I}) \to \mathcal{L}(\mathcal{H}^{A_O} \otimes \mathcal{H}^{A'_O})$ and $B : \mathcal{L}(\mathcal{H}^{B_I} \otimes \mathcal{H}^{B'_I}) \to \mathcal{L}(\mathcal{H}^{B_O} \otimes \mathcal{H}^{B'_O})$, where the dimension of the primed spaces is arbitrary. This results in the quantum channel $\mathcal{S} \otimes \mathcal{I}(A, B) : \mathcal{L}(\mathcal{H}^{A'_I} \otimes \mathcal{H}^{c_I} \otimes \mathcal{H}^{t_I} \otimes \mathcal{H}^{B'_I}) \to \mathcal{L}(\mathcal{H}^{A'_O} \otimes \mathcal{H}^{c_O} \otimes \mathcal{H}^{t_O} \otimes \mathcal{H}^{B'_O})$, where $\mathcal{I}$ is the identity higher-order transformation. Since the dimension of the primed spaces is arbitrary, all multi-partite channels can be described in this context as bipartite channels, which are the most general kinds of deterministic transformations between quantum states one can consider. This case is illustrated in Fig. 3 of the main text.

As mentioned in the main text, the impossibility of simulating the action of the quantum switch on all single-party quantum channels, for some number of calls k_A and k_B, implies the impossibility of simulating the action of the quantum switch on all bipartite channels with the same number of calls. Hence, when focusing on no-go simulation proofs, we can restrict ourselves to considering only single-party channels. However, conversely, should a simulation of the action of the quantum switch on all single-party channels exist for some number of calls k_A and k_B, this does not necessarily imply that a simulation would also exists for the action of the quantum switch on all bipartite channels.

For this reason, in order to have a fully general simulation of the quantum switch, one must consider the case where the quantum switch acts on only part of a bipartite channel, as illustrated in Fig. 1. That is, a full simulation of the quantum switch is only obtained when, for every pair of bipartite channels $A : \mathcal{L}(\mathcal{H}^{A_I} \otimes \mathcal{H}^{A'_I}) \to \mathcal{L}(\mathcal{H}^{A_O} \otimes \mathcal{H}^{A'_O})$ and $B : \mathcal{L}(\mathcal{H}^{B_I} \otimes \mathcal{H}^{B'_I}) \to \mathcal{L}(\mathcal{H}^{B_O} \otimes \mathcal{H}^{B'_O})$, there exists $k_A, k_B \in \mathbb{N}$ such that,

$$\mathcal{C}(A^{\otimes k_A}, B^{\otimes k_B}) = \mathcal{S} \otimes \mathcal{I}(A, B) \tag{32}$$

where $\mathcal{C}$ is a quantum comb, or a QC-CC. This most general simulation scenario is depicted in Fig. 1.

In the next section, we show how a simulation of the action of the quantum switch on all pairs of bipartite channels indeed covers all possible quantum operations the quantum switch could take as input, including the probabilistic ones, such as quantum instruments.

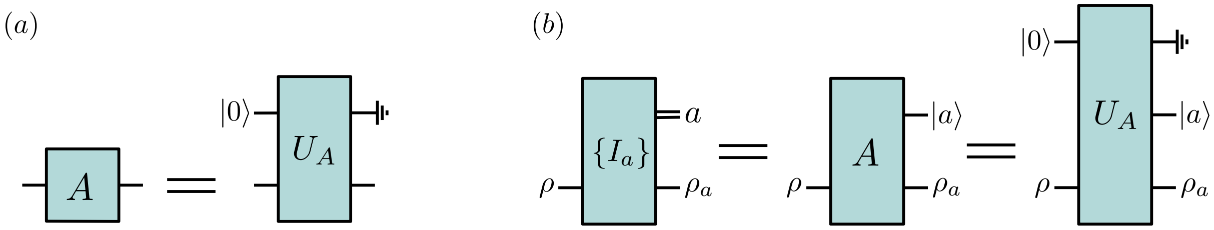

Supplementary Figure 2. **General quantum channels and quantum instruments.** (a) Quantum circuit representation of the Stinespring dilation U_A of the general quantum channel A presented in Eq. (34). (b) Quantum circuit representation of a quantum instrument $\{I_a\}_a$ by a quantum channel $A[\rho] := \sum_a I_a[\rho] \otimes |a\rangle\langle a|$, which is then represented by its Stinespring dilation U_A.

2.2 The action of the quantum switch on quantum instruments

Higher-order transformations like the quantum switch can also be applied to quantum instruments, which describe the most general probabilistic transformation between quantum states.

Quantum instruments are transformations that map a quantum state to another quantum state with a certain probability, also outputting a classical outcome. Physically, this describes, for instance, a measurement process where both a classical outcome and a post-measurement quantum state are produced. They can be described by a collection of CP maps $\{I_a\}_a$ which add to a CPTP map, i.e., such that $\sum_a I_a$ is CPTP. A quantum instrument takes a quantum state ρ as input, and outputs a classical outcome a, together with the quantum state $\rho_a := I_a[\rho]/\mathrm{tr}(I_a[\rho])$, with probability $p_a := \mathrm{tr}(I_a[\rho])$. Quantum instruments can be equivalently represented by deterministic quantum channels, which instead of yielding a classical outcome, prepare an additional output pure quantum state $|a\rangle$ that encodes the value of the instrument's classical outcome a in a perfectly discriminable manner [4–6]. In other words, a quantum instrument with $N \in \mathbb{N}$ outcomes $\{I_a\}_{a=1}^N$ with $I_a : \mathcal{L}(\mathcal{H}^I) \to \mathcal{H}(\mathcal{H}^O)$ is equivalent to a quantum channel $A : \mathcal{L}(\mathcal{H}^I) \to \mathcal{H}(\mathcal{H}^O \otimes \mathbb{C}^N)$ given by

$$A[\,\rho\,] := \sum_{a=1}^{N} I_a[\,\rho\,] \otimes |a\rangle\langle a|\,, \tag{33}$$

and illustrated in Fig. 2(b). By representing a quantum instrument as a particular case of a bipartite quantum channel, namely one with a single input system and two output systems, it is straightforward to see that the action of the quantum switch on a pair of instruments $\{I_a^A\}_a$ and $\{I_b^B\}_b$ constitutes a special case of its action on arbitrary bipartite channels A and B. Hence, a simulation of the quantum switch for all bipartite channels can be used for a simulation of the quantum switch for all instruments.

Lastly, one might also consider the scenario where the quantum switch acts on a part of bipartite instruments. Notice, however, that from the channel representation of instruments, it is clear that this scenario is included in that of arbitrary bipartite channels discussed in the previous section.

2.3 The relationship with Stinespring dilation of quantum channels

The Stinespring dilation theorem [7] states that the action of every general quantum channel, i.e, CPTP map, $A : \mathcal{L}(\mathcal{H}^I) \to \mathcal{L}(\mathcal{H}^O)$ can be written as

$$A[\,\rho\,] = \mathrm{tr}_{\mathrm{aux}_O}\Big(U_A[\,\rho^I \otimes |0\rangle\langle 0|\,]\Big), \tag{34}$$

for some unitary channel $U_A : \mathcal{L}(\mathcal{H}^I \otimes \mathcal{H}^{\mathrm{aux}_I}) \to \mathcal{L}(\mathcal{H}^O \otimes \mathcal{H}^{\mathrm{aux}_O})$ that acts jointly on the input system and an auxiliary system (of sufficiently large dimension), which can be initialised to the state $|0\rangle$ without loss of generality. We emphasise that the discarding of the output auxiliary system is necessary for the equivalence between non-unitary channels and their unitary channel dilation. This equivalence between arbitrary quantum channels and their unitary

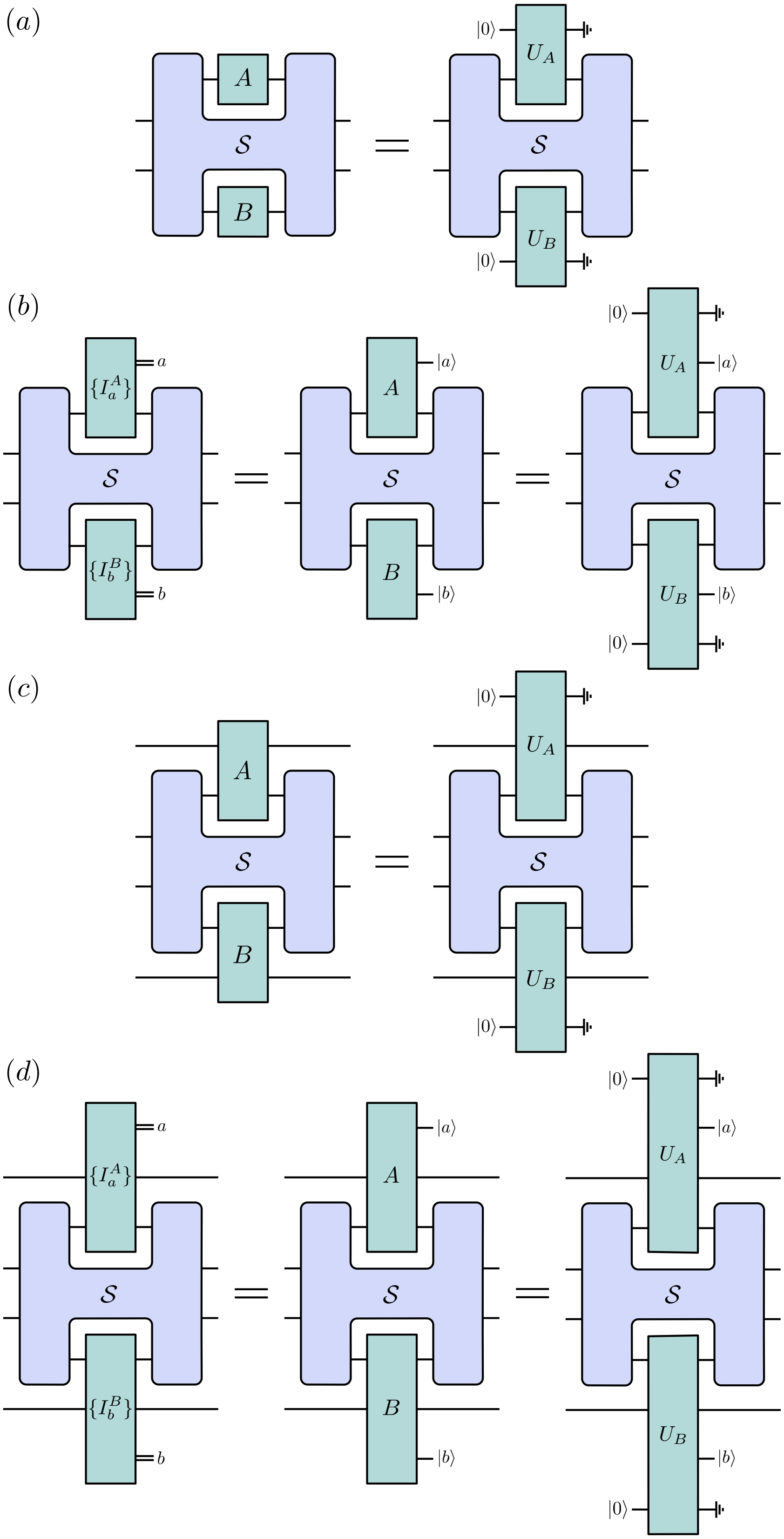

Supplementary Figure 3. **The action of the quantum switch.** Depiction of the action of the quantum switch on (a) general single-party quantum channels; (b) general single-party quantum instruments; (c) part of general bipartite quantum channels, and; (d) part of general bipartite quantum instruments; as a function of the action of the quantum switch on the Stinespring dilation of the input channels.

channel dilation is depicted in Fig. 2(a). Additionally, note that the Stinespring dilation can be combined with the channel representation of a quantum instrument presented in Eq. (33), so that all instruments can also be viewed as a unitary channel that makes use of an auxiliary system which is later discarded, as depicted in Fig. 2(b).

Exploiting the Stinespring dilation of general quantum instruments and general quantum channels into bipartite unitary channels that discard their auxiliary system, one can express the action—and consequently the simulation—of the quantum switch solely in terms of unitary channels. However, in order to do so, it is necessary to keep track of exactly which systems are acted upon, preserved, and discarded. In Fig. 3, we show precisely how the action of the quantum switch on the different kinds of inputs discussed so far can be expressed in terms of its action on unitary channels.

In Fig. 3(a), we depict the action of the quantum switch on general single-party channels. In Fig. 3(b), we represent the action of the quantum switch on single-party quantum instruments. In Fig. 3(c), we show the action of the switch in its most general case: when it acts on only part of bipartite general channels. Finally, in Fig. 3(d), the action of the switch on part of bipartite instruments is pictured. Trivially, the case in Fig. 3(a) is a particular case of Fig. 3(c). Notice, moreover, how both the cases of Fig. 3(b) and (d), concerning quantum instruments, are also particular cases of Fig. 3(c). In the latter case in particular, this is because the dimension of the primed spaces of the input bipartite channels—the ones that the switch does not act upon—are arbitrary, and hence, the quantum outputs $|a\rangle$ and $|b\rangle$ of the instruments can be absorbed into the other output system of the unitary channels that is not acted upon by the quantum switch.

2.4 Theorem 1: A simulation for bipartite unitary channels U_A and bipartite general channels B

In this section, we prove Theorem 1 from the main text and discuss how this particular-case simulation compares to more general simulation scenarios. We start by reproducing Eq. (8) of the main text which defines a general simulation of the quantum switch. Namely, a simulation $\mathcal{C}$ that is able to prepare, with some finite number of calls k_A and k_B, a channel $\mathcal{C}(A^{\otimes k_A}, B^{\otimes k_B})$ such that

$$\mathcal{C}(A^{\otimes k_A}, B^{\otimes k_B}) = \mathcal{S} \otimes \mathcal{I}(A, B) \quad \forall\ A, B, \tag{35}$$

where A, B are arbitrary quantum channels, is a higher-order transformation that can simulate the action of the quantum switch in its most general form.

We are now ready to restate and prove Theorem 1:

Theorem 1. *The action of the quantum switch on part of bipartite quantum channels can be deterministically simulated by a quantum circuit that has access to an extra call to one the input channels, as long as that channel is restricted to being unitary.*

In other words, if A is a bipartite unitary channel and B is a bipartite general channel, there exists a quantum circuit described by a higher-order transformation $\mathcal{C}$ that satisfies Eq. (35) for $k_A = 2$ and $k_B = 1$.

The proof is based on the explicit construction of the following quantum circuit, presented as Eq. (9) in the main text and repeated here for convenience:

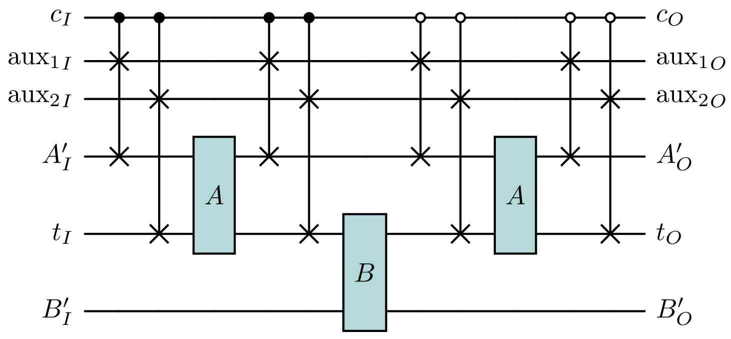

(36)

Proof. Let $A : \mathcal{L}(\mathcal{H}^{A_I} \otimes \mathcal{H}^{A'_I}) \to \mathcal{L}(\mathcal{H}^{A_O} \otimes \mathcal{H}^{A'_O})$ be a bipartite channel with Kraus operators $\{\mathsf{A}_i\}$ where $\mathsf{A}_i : \mathcal{H}^{A_I} \otimes \mathcal{H}^{A'_I} \to \mathcal{H}^{A_O} \otimes \mathcal{H}^{A'_O}$. Similarly, let $B : \mathcal{L}(\mathcal{H}^{B_I} \otimes \mathcal{H}^{B'_I}) \to \mathcal{L}(\mathcal{H}^{B_O} \otimes \mathcal{H}^{B'_O})$ be a bipartite channel with Kraus operators $\{\mathsf{B}_j\}$ where $\mathsf{B}_j : \mathcal{H}^{B_I} \otimes \mathcal{H}^{B'_I} \to \mathcal{H}^{B_O} \otimes \mathcal{H}^{B'_O}$. The action of the quantum switch $\mathcal{S}$ on part of these bipartite channels results in the quantum channel $\mathcal{S} \otimes \mathcal{I}(A, B) : \mathcal{L}(\mathcal{H}^{c_I} \otimes \mathcal{H}^{t_I} \otimes \mathcal{H}^{A'_I} \otimes \mathcal{H}^{B'_I}) \to \mathcal{L}(\mathcal{H}^{c_O} \otimes \mathcal{H}^{t_O} \otimes \mathcal{H}^{A'_O} \otimes \mathcal{H}^{B'_O})$, whose definition is implied by the action of the quantum switch on single-party channels and linearity. It is given by

$$\mathcal{S} \otimes \mathcal{I}(A, B)[\,\sigma_c \otimes \rho_t \otimes \omega_{A'B'}\,] = \sum_{i,j} \mathsf{S}_{ij}(\sigma_c \otimes \rho_t \otimes \omega_{A'B'})\mathsf{S}_{ij}^\dagger, \tag{37}$$

where $\omega_{A'B'} \in \mathcal{L}(\mathcal{H}^{A'_I} \otimes \mathcal{H}^{B'_I})$ is an arbitrary state of the quantum system in the primed spaces of Alice and Bob, which are not acted upon by the quantum switch. Before explicitly writing the S_{ij}, it is convenient to decompose the Kraus operators A_i and B_i into linear combinations of operators that factorise between the primed and nonprimed spaces, which can always be done without loss of generality. It follows from linearity that one can always write

$$\mathsf{A}_i = \sum_a \alpha_{a|i}\, \mathsf{A}(a) \otimes \mathsf{A}'(a) \tag{38}$$

for some $\alpha_{a|i} \in \mathbb{C}$, $\mathsf{A}(a) : \mathcal{H}^{A_I} \to \mathcal{H}^{A_O}$, and $\mathsf{A}'(a) : \mathcal{H}^{A'_I} \to \mathcal{H}^{A'_O}$. Similarly, one can write

$$\mathsf{B}_j = \sum_b \beta_{b|j}\, \mathsf{B}(b) \otimes \mathsf{B}'(b) \tag{39}$$

for some $\beta_{b|j} \in \mathbb{C}$, $\mathsf{B}(b) : \mathcal{H}^{B_I} \to \mathcal{H}^{B_O}$, and $\mathsf{B}'(b) : \mathcal{H}^{B'_I} \to \mathcal{H}^{B'_O}$. Then, the Kraus operators $\{\mathsf{S}_{ij}\}$ in Eq. (37) take the form

$$\mathsf{S}_{ij} = |0\rangle\langle 0| \otimes \sum_{ab} \alpha_{a|i}\, \beta_{b|j}\, \mathsf{B}(b)\mathsf{A}(a) \otimes \mathsf{A}'(a) \otimes \mathsf{B}'(b) + |1\rangle\langle 1| \otimes \sum_{ab} \alpha_{a|i}\, \beta_{b|j}\, \mathsf{A}(a)\mathsf{B}(b) \otimes \mathsf{A}'(a) \otimes \mathsf{B}'(b), \tag{40}$$

where the order of the spaces from left to right in each term is control, target, and Alice and Bob's primed systems.

The transformation of the quantum circuit in Eq. (36) that uses two calls of the quantum channel A and a single call of the quantum channel B in the order ABA results in a quantum channel $\mathcal{C}(A^{\otimes 2}, B)$ which has Kraus operators

$$\mathsf{C}_{ijk} = |0\rangle\langle 0| \otimes \mathsf{A}_k \otimes \sum_{ab} \alpha_{a|i}\, \beta_{b|j}\, \mathsf{A}'(a) \otimes \mathsf{B}(b)\mathsf{A}(a) \otimes \mathsf{B}'(b) + |1\rangle\langle 1| \otimes \mathsf{A}_i \otimes \sum_{ab} \alpha_{a|k}\, \beta_{b|j} \mathsf{A}'(a) \otimes \mathsf{A}(a)\mathsf{B}(b) \otimes \mathsf{B}'(b), \tag{41}$$

where the order of the spaces from left to right in each term coincides with the order of the wires from top to bottom in the quantum circuit in Eq. (36) (i.e., control, first auxiliary, second auxiliary, Alice's primed, target, and Bob's primed systems).

Whenever the bipartite channel $A = U_A$ is unitary, it has a single Kraus operator, i.e., $A[\rho] = \mathsf{A}_0 \rho \mathsf{A}_0^\dagger$. Hence, following Eq. (40), in this case the quantum channel resulting from action of the quantum switch $\mathcal{S} \otimes \mathcal{I}(U_A, B)$ has Kraus operators

$$\mathsf{S}_j = |0\rangle\langle 0| \otimes \sum_{ab} \alpha_{a|0}\, \beta_{b|j}\, \mathsf{B}(b)\mathsf{A}(a) \otimes \mathsf{A}'(a) \otimes \mathsf{B}'(b) + |1\rangle\langle 1| \otimes \sum_{ab} \alpha_{a|0}\, \beta_{b|j}\, \mathsf{A}(a)\mathsf{B}(b) \otimes \mathsf{A}'(a) \otimes \mathsf{B}'(b). \tag{42}$$

Also in this case, following Eq. (41), the quantum channel resulting from action of the quantum circuit in Eq. (36) is described by the Kraus operators

$$\mathsf{C}_j = |0\rangle\langle 0| \otimes \mathsf{A}_0 \otimes \sum_{ab} \alpha_{a|0}\, \beta_{b|j}\, \mathsf{A}'(a) \otimes \mathsf{B}(b)\mathsf{A}(a) \otimes \mathsf{B}'(b) + |1\rangle\langle 1| \otimes \mathsf{A}_0 \otimes \sum_{ab} \alpha_{a|0}\, \beta_{b|j}\, \mathsf{A}'(a) \otimes \mathsf{A}(a)\mathsf{B}(b) \otimes \mathsf{B}'(b) \tag{43}$$

$$= \left(|0\rangle\langle 0| \otimes \sum_{ab} \alpha_{a|0}\, \beta_{b|j}\, \mathsf{B}(b)\mathsf{A}(a) \otimes \mathsf{A}'(a) \otimes \mathsf{B}'(b) + |1\rangle\langle 1| \otimes \sum_{ab} \alpha_{a|0}\, \beta_{b|j}\, \mathsf{A}(a)\mathsf{B}(b) \otimes \mathsf{A}'(a) \otimes \mathsf{B}'(b) \right) \otimes \mathsf{A}_0. \tag{44}$$

where, in the last line, we reordered the spaces from control, first auxiliary, second auxiliary, Alice's primed, target, and Bob's primed systems to control, target, and Alice and Bob's primed, first auxiliary, and second auxiliary systems.

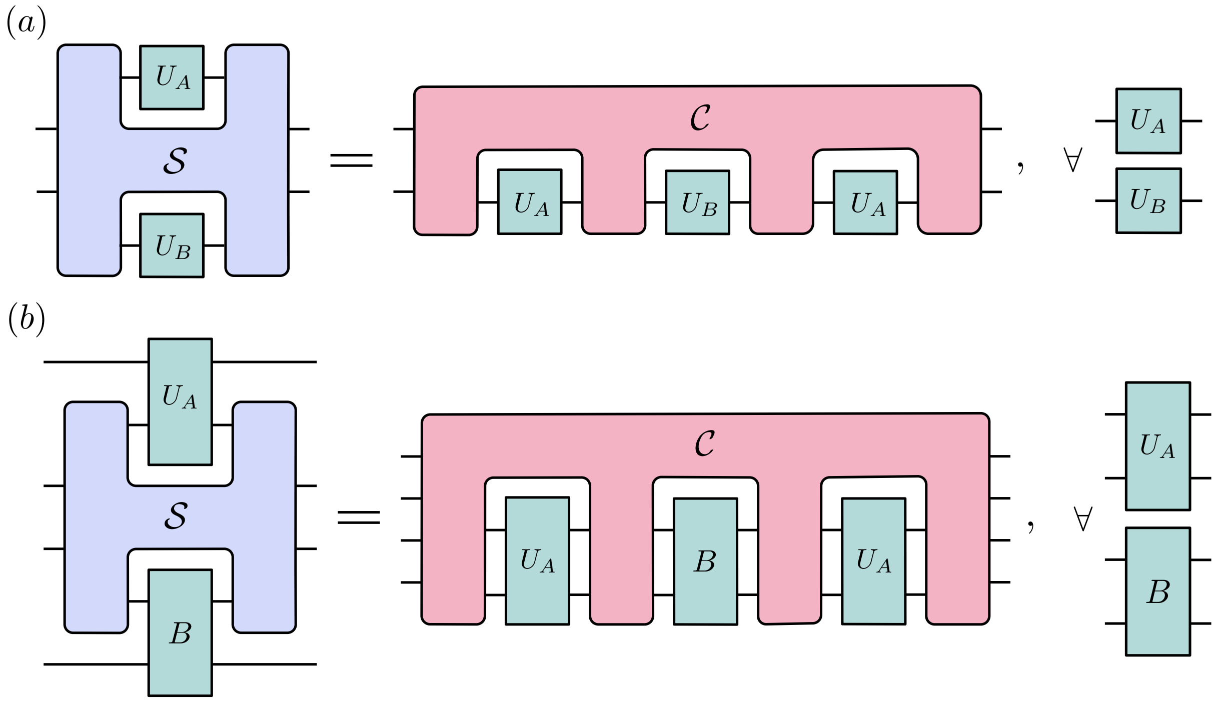

Supplementary Figure 4. **Quantum comb representation of possible quantum switch simulations.** (a) Representation of the simulation of the action of the quantum switch on single-party unitary channels as a quantum comb, presented in Ref. [9] and reproduced in the main text as the quantum circuit in Eq. (6). (b) Representation of the simulation of the action of the quantum switch on part of a bipartite unitary channel and part of a bipartite general quantum channel by a quantum comb, which we have shown to be possible by explicitly constructing the quantum circuit in Eq. (36) in Thm. 1.

Note that the term in parenthesis is equal to that of Eq. (42). Hence, the action of the quantum switch on part of a bipartite unitary channel $A = U_A$ and a bipartite general channel B is equivalent to the action of the quantum circuit in Eq. (36) that uses two calls of bipartite unitary channel $A = U_A$ and a single call of bipartite general channel B in the order ABA. Moreover, note that one call of $A = U_A$ in the quantum circuit in Eq. (36) is recovered, since A_0 factorizes in Eq. (43). This phenomenon is referred to as a catalytic higher-order transformation in the literature [8], since the extra use of $A = U_A$ is recovered after the completion of the process. □

We now contrast the different scenarios of the action of the quantum switch presented in Secs. 2 2.1–2 2.3 with the particular-case simulation of Theorem 1.

Let us begin with the circuit from Ref. [9], reproduced in Eq. (6) of the main text, and depicted here as the quantum comb in Fig. 4(a). This circuit simulates the action of the quantum switch on unitary channels by making use of one extra call to one of the input channels. Although every general quantum channel can be dilated into a unitary channel acting on a larger space, the simulation of the quantum switch on unitary channels in the case depicted in Fig. 4(a) is not the most general case, because here the *entire* unitary channel is "plugged into" the open slots of the quantum switch and of the quantum comb that performs its simulation.

The simulation presented in Ref. [9], here in Fig. 4(a), is a particular case of the simulation scenario we proved possible via the explicit construction of the quantum circuit in Eq. (36). Our generalization addresses the case where the quantum switch acts on only part of a unitary channel and part of a general quantum channel, with the simulation also requiring only one extra copy of the input unitary channel. We depict this scenario and our quantum circuit simulation as a quantum comb in Fig. 4(b). Although more general than the previous result from Ref. [9], this simulation result still does not hold in the most general case, depicted in Fig. 1(c), because the input quantum channel U_A is required to be a unitary channel.

The quantum switch simulation presented in Fig. 4(b) is also useful to illustrate the crucial aspect of the partial trace involved in the Stinespring dilation. Notice that this simulation covers the scenario where B is an arbitrary bipartite channel, and U_A is an arbitrary bipartite unitary channel. Naïvely, one could expect that—since our simulation covers

all bipartite unitary channels U_A—due to the Stinespring dilation theorem, this simulation would also apply to the case where A is a single-party arbitrary quantum channel, as in Fig. 3(a). However, this line of argumentation is false, as it is not possible to simulate the quantum switch for a pair of single-party arbitrary quantum channels, as proven in the main text. The logical gap in the argument is a misuse of the Stinespring dilation theorem, which requires the auxiliary system to be discarded, whereas in the simulation presented in Fig. 4(b), there is no partial trace after the unitary operation U_A. When the auxiliary system of the Stinespring dilation is not discarded, the corresponding operation is not equivalent to the quantum channel in question. From this circuit simulation perspective, keeping track of the auxiliary systems may be viewed as a "loophole", since the auxiliary system may carry additional information that is not provided by the channel A. To see this intuitively, consider a quantum channel A with a Kraus decomposition given by $A[\rho] = \sum_i \mathsf{K}_i \rho \mathsf{K}_i^\dagger$ and which is dilated by the isometry $\mathsf{V} = \sum_i \mathsf{K}_i \otimes |i\rangle$, where $|i\rangle$ is a quantum state on the auxiliary system. The quantum state $|i\rangle$ may be viewed as a flag that indicates which Kraus operator was applied to ρ. If the flag state is not discarded, one could use this information to correlate Kraus elements between a first and a second call of the channel A, which cannot be done when the auxiliary system is traced out.

Supplementary Note 3 – Full proof of Theorem 2

This section alone will adopt a slightly different notation from the main text and the remainder of the SI. This is due to the fact that the proof technique of this particular theorem is different from those applied to prove and present the other results, and it benefits from a different notation. Below is a summary of the changes, to facilitate the transition into this next section:

- We will denote a Hilbert space simply by the label of the system it describes, according to $X \coloneqq \mathcal{H}^X$. In particular, the input and output Hilbert spaces of the control and target systems in the quantum switch higher-order transformation will be denoted instead as "past" and "future", using the notation $P_C \coloneqq \mathcal{H}^{c_I}$, $P_T \coloneqq \mathcal{H}^{t_I}$, $F_C \coloneqq \mathcal{H}^{c_O}$, $F_T \coloneqq \mathcal{H}^{t_O}$, and collectively as $P \coloneqq P_C \otimes P_T$ and $F \coloneqq F_C \otimes F_T$.

- Quantum channels will be denoted by cursive letters $\mathcal{A}$ and $\mathcal{B}$ instead of A and B. Kraus operators and unitary operators will be denoted by A_i, B_j and U, V instead of A_i, B_j and U, V. Higher-order transformations will continue to be denoted with cursive letters, such as $\mathcal{S}$ for the quantum switch and $\mathcal{C}$ for quantum combs or QC-CC simulators, as in the remainder of the SI.

- Higher-order transformations that correspond to the simulator, be it a quantum comb or a QC-CC, will be denoted as a function $\mathcal{C}$ that has as many arguments as there are slots in the transformation (e.g., $\mathcal{C} = \mathcal{C}(\mathcal{A}, \mathcal{B}, \mathcal{C})$ for a 3-slot higher-order transformation). The number of available calls to channel $\mathcal{A}$ and $\mathcal{B}$ will be denoted as M (instead of k_A) and N (instead of k_B). Hence, what would be written as $\mathcal{C}(A^{\otimes k_A}, B^{\otimes k_B})$ in the remainder of the SI, in this section becomes instead $\mathcal{C}(\underbrace{\mathcal{A}, \ldots, \mathcal{A}}_{M}, \underbrace{\mathcal{B}, \ldots, \mathcal{B}}_{N})$.

- Choi operators, Choi vectors, and the link product will be used as defined in Sec. IV Methods of the main text.

We are now ready to restate and prove Thm. 2.

Theorem 2 (expanded). *Let $\mathcal{S} : [\mathcal{L}(I) \to \mathcal{L}(O)] \otimes [\mathcal{L}(I') \to \mathcal{L}(O')] \to [\mathcal{L}(P) \to \mathcal{L}(F)]$, where I, O, I', O' are n-qubit Hilbert spaces and P, F are $(n+1)$-qubit Hilbert spaces, be the quantum switch.*

There is no $(M+1)$-slot quantum circuit with classical control of causal order (QC-CC) higher-order transformation $\mathcal{C} : \bigotimes_{i=1}^{M} [\mathcal{L}(I_i) \to \mathcal{L}(O_i)] \otimes [\mathcal{L}(I'_1) \to \mathcal{L}(O'_1)] \to [\mathcal{L}(P) \to \mathcal{L}(F)]$, where $\{I_i\}_i, \{O_i\}_i, I'_1, O'_1$ are n-qubit Hilbert spaces, satisfying

$$\mathcal{C}(\underbrace{\mathcal{A}, \ldots, \mathcal{A}}_{M}, \mathcal{B}) = \mathcal{S}(\mathcal{A}, \mathcal{B}) \tag{45}$$

for all mixed unitary channels $\mathcal{A}$ and unitary channels $\mathcal{B}$, if $M \le \max(2, 2^n - 1)$.

Proof. The proof is based upon a series of lemmas, proven below. First, we assume that $\mathcal{C}$ is a multilinear higher-order transformation whose Choi operator C is positive semidefinite, and hence can be written as

$$C = \sum_a |C^{(a)}\rangle\!\rangle\langle\!\langle C^{(a)}| \tag{46}$$

with $|C^{(a)}\rangle\!\rangle\langle\!\langle C^{(a)}| \geq 0$ for all a.

Let us write the mixed unitary channel $\mathcal{A}$ in Eq. (45) as $\mathcal{A} = \frac{1}{M}\sum_{i=1}^{M} \mathcal{A}_i$, for some $M \in \mathbb{N}^+$, where $\{\mathcal{A}_i\}_i$ are unitary channels with associated unitary operators $\{U_i\}_i$, and let unitary channel $\mathcal{B}$ be associated to unitary operator V_1. We then invoke Lemma 2 (which requires Lemma 1 for its proof) for $N = 1$ to say that, since it is assumed that $\mathcal{C}$ satisfies Eq. (45), each $|C^{(a)}\rangle\!\rangle\langle\!\langle C^{(a)}|$ satisfies

$$|C^{(a)}\rangle\!\rangle * \Big(\bigotimes_{k=1}^{M} |U_k\rangle\!\rangle \otimes |V_1\rangle\!\rangle\Big) = \sum_{k=1}^{M} \xi_{k1}^{(a)}(\{U_i\}_i,\ V_1)\, |S\rangle\!\rangle * \Big(|U_k\rangle\!\rangle \otimes |V_1\rangle\!\rangle\Big) \tag{47}$$

for some coefficients $\xi_{k1}^{(a)}(\{U_i\}_i,\ V_1) \in \mathbb{C}$, where $|S\rangle\!\rangle$ is the Choi vector of the quantum switch.

From Lemma 3, we know that if there exist coefficients $\xi_{k1}^{(a)} \leftarrow \tilde{\xi}_{k1}^{(a)}$ such that, for all $k \in \{1, \ldots, M\}$, $\tilde{\xi}_{k1}^{(a)}$ is simultaneously

1. independent of U_k and V_1, and

2. linear in $U_{k'}$ for all $k' \neq k$,

then, there exist vectors $|\tilde{\xi}^{(a)}\rangle\!\rangle^{\{I_1O_1,\cdots,I_MO_M\}\backslash\{I_kO_k\}}$ independent of $\{U_i\}_i$ and V_1 such that

$$|C^{(a)}\rangle\!\rangle^{PI_1O_1\cdots I_MO_MI_1'O_1'F} = \sum_{k=1}^{M} |S\rangle\!\rangle^{PI_kO_kI_1'O_1'F} \otimes |\tilde{\xi}_{k1}^{(a)}\rangle\!\rangle^{\{I_1O_1,\cdots,I_MO_M\}\backslash\{I_kO_k\}} \tag{48}$$

for all a. Such conditions are guaranteed to be satisfied via Lemma 4 when $M < 4^n/2 + 2$ [which is implied by $M \leq \max(2, 2^n - 1)$].

Finally, invoking Lemma 5 (which requires Lemmas 6 and 7 for its proof), we find that if $M \leq \max(2, 2^n - 1)$, a higher-order transformation with Choi operator $C = \sum_a |C^{(a)}\rangle\!\rangle\langle\!\langle C^{(a)}|$, where $|C^{(a)}\rangle\!\rangle$ satisfies Eqs. (48) for all a does not satisfy the conditions of a QC-CC transformation. □

3.1 Lemma 1

Lemma 1. *Let $|\phi\rangle$ and $\{|\psi_i\rangle\}_i$ be vectors in $\mathbb{C}^d$.*

$$\textit{If } |\phi\rangle\langle\phi| \leq \sum_i |\psi_i\rangle\langle\psi_i|\,,\ \textit{then}\, |\phi\rangle \in \mathrm{span}(\{|\psi_i\rangle\}). \tag{49}$$

That is, there exist complex numbers α_i such that $|\phi\rangle = \sum_i \alpha_i |\psi_i\rangle$.

Proof. The proof will go by contradiction. We start by pointing out that any vector $|\phi\rangle \in \mathbb{C}^d$ can be decomposed as

$$|\phi\rangle = |\psi\rangle + |\psi_\perp\rangle\,, \tag{50}$$

where $|\psi\rangle \in \mathrm{span}(\{|\psi_i\rangle\})$, $|\psi_\perp\rangle \notin \mathrm{span}(\{|\psi_i\rangle\})$. Also, since $|\psi_\perp\rangle \notin \mathrm{span}(\{|\psi_i\rangle\})$, we have that $\langle\psi_\perp|\psi_i\rangle = 0$ for every i.

Now, assume that $|\phi\rangle \notin \mathrm{span}(\{|\psi_i\rangle\})$. In this case, we necessarily have that $|\psi_\perp\rangle \neq 0$. Using this decomposition $|\phi\rangle = |\psi\rangle + |\psi_\perp\rangle$, we can write the inequality $|\phi\rangle\langle\phi| \leq \sum_i |\psi_i\rangle\langle\psi_i|$, as

$$|\psi\rangle\langle\psi| + |\psi\rangle\langle\psi_\perp| + |\psi_\perp\rangle\langle\psi| + |\psi_\perp\rangle\langle\psi_\perp| \leq \sum_i |\psi_i\rangle\langle\psi_i|\,. \tag{51}$$

We then apply $\langle\psi_\perp|$ and $|\psi_\perp\rangle$ on both sides of operator inequality in Eq. (51) to obtain the real number inequality

$$\langle\psi_\perp|\psi_\perp\rangle\,\langle\psi_\perp|\psi_\perp\rangle \leq 0. \tag{52}$$

However, since $|\psi_\perp\rangle \neq 0$, $\langle\psi_\perp|\psi_\perp\rangle\,\langle\psi_\perp|\psi_\perp\rangle$ is strictly positive, hence we have arrived at a contradiction. Therefore, $|\phi\rangle$ must belong to the span($\{|\psi_i\rangle\}$). □

3.2 Lemma 2 (for $M, N \in \mathbb{N}^+$)

Lemma 2. *Let $\mathcal{S} : [\mathcal{L}(I) \to \mathcal{L}(O)] \otimes [\mathcal{L}(I') \to \mathcal{L}(O')] \to [\mathcal{L}(P) \to \mathcal{L}(F)]$, where I, O, I', O' correspond to d-dimensional Hilbert spaces and P, F correspond to $(2 \times d)$-dimensional Hilbert spaces, be a multilinear higher-order transformation that acts on two quantum channels and has a rank-1 Choi operator $S = |S\rangle\!\rangle\langle\!\langle S|$ (such as, e.g., the quantum switch). Furthermore, let $\mathcal{C} : \bigotimes_{i=1}^M [\mathcal{L}(I_i) \to \mathcal{L}(O_i)] \otimes \bigotimes_{j=1}^N [\mathcal{L}(I'_j) \to \mathcal{L}(O'_j)] \to [\mathcal{L}(P) \to \mathcal{L}(F)]$, where $M, N \in \mathbb{N}^+$ and $\{I_i\}_i, \{O_i\}_i, \{I'_j\}_j, \{O'_j\}_j$ correspond to d-dimensional Hilbert spaces, be a multilinear higher-order transformation that acts on $M + N$ quantum channels with a positive semidefinite Choi operator C, which, therefore, can be written as*

$$C = \sum_a |C^{(a)}\rangle\!\rangle\langle\!\langle C^{(a)}| \,, \tag{53}$$

where each $|C^{(a)}\rangle\!\rangle\langle\!\langle C^{(a)}| \geq 0$. Finally, let $\mathcal{A}$ and $\mathcal{B}$ be mixed unitary channels, such that $\mathcal{A} = \frac{1}{K}\sum_{i=1}^K \mathcal{A}_i$ and $\mathcal{B} = \frac{1}{L}\sum_{j=1}^L \mathcal{B}_j$ for some $K, L \in \mathbb{N}^+$, where $\{\mathcal{A}_i\}_i$ and $\{\mathcal{B}_j\}_j$ are unitary channels with associated unitary operators $\{U_i\}_i$ and $\{V_j\}_j$, respectively.

If $\mathcal{C}$ corresponds to a simulation of the action of $\mathcal{S}$ on mixed unitary channels $\mathcal{A}$ and $\mathcal{B}$, i.e., if $\mathcal{C}$ satisfies

$$\mathcal{C}(\underbrace{\mathcal{A}, \ldots, \mathcal{A}}_{M}, \underbrace{\mathcal{B}, \ldots, \mathcal{B}}_{N}) = \mathcal{S}(\mathcal{A}, \mathcal{B}) \tag{54}$$

for all mixed unitary channels $\mathcal{A}$ and $\mathcal{B}$ (or, if $N = 1$, for all mixed unitary channels $\mathcal{A}$ and unitary channels $\mathcal{B}$), then, for every a, there exist coefficients $\{\xi^{(a)}_{kl}(\{U_i\}_i,\ \{V_j\}_j)\}_{kl} \in \mathbb{C}$ such that

$$|C^{(a)}\rangle\!\rangle * \big(|U_1\rangle\!\rangle \otimes \ldots \otimes |U_M\rangle\!\rangle \otimes |V_1\rangle\!\rangle \otimes \ldots \otimes |V_N\rangle\!\rangle\big) = \sum_{k=1}^{M}\sum_{l=1}^{N} \xi^{(a)}_{kl}(\{U_i\}_i,\ \{V_j\}_j)|S\rangle\!\rangle * \big(|U_k\rangle\!\rangle \otimes |V_l\rangle\!\rangle\big) \,. \tag{55}$$

Proof. Since, by assumption, Eq. (54) holds for all mixed unitary channels $\mathcal{A}, \mathcal{B}$, for any sets of unitary channels $\{\mathcal{A}_1, \ldots \mathcal{A}_K\}$ and $\{\mathcal{B}_1, \ldots \mathcal{B}_L\}$ with $K, L \geq 1$, one has that

$$\mathcal{C}\left(\sum_{i_1=1}^K \frac{\mathcal{A}_{i_1}}{K}, \ldots, \sum_{i_M=1}^K \frac{\mathcal{A}_{i_M}}{K}; \sum_{j_1=1}^L \frac{\mathcal{B}_{j_1}}{L}, \ldots, \sum_{j_N=1}^L \frac{\mathcal{B}_{j_N}}{L}\right) = \mathcal{S}\left(\sum_{k=1}^K \frac{\mathcal{A}_k}{K}, \sum_{l=1}^L \frac{\mathcal{B}_l}{L}\right) \,. \tag{56}$$

Note that for $N = 1$, it is sufficient to assume that Eq. (54) holds for all mixed unitary channels $\mathcal{A}$ and unitary channels $\mathcal{B}$, in which case we take $L = 1$.

From the multilinearity of $\mathcal{C}$ and $\mathcal{S}$, it follows that

$$\frac{1}{K^M L^N} \sum_{i_1,\ldots,i_M=1}^K \sum_{j_1,\ldots,j_N=1}^L \mathcal{C}\left(\mathcal{A}_{i_1}, \ldots, \mathcal{A}_{i_M}; \mathcal{B}_{j_1}, \ldots, \mathcal{B}_{j_N}\right) = \frac{1}{KL}\sum_{k=1}^K\sum_{l=1}^L \mathcal{S}\left(\mathcal{A}_k, \mathcal{B}_l\right) \,. \tag{57}$$

Rewriting this expression in the Choi representation—using the convention that C, S, A_i, B_j are the Choi matrices of

$\mathcal{C}, \mathcal{S}, \mathcal{A}_i, \mathcal{B}_j$, respectively—gives

$$\sum_{i_1,\ldots,i_M=1}^{K}\sum_{j_1,\ldots,j_N=1}^{L} C * \left(A_{i_1}\otimes\cdots\otimes A_{i_M}\otimes B_{j_1}\otimes\cdots\otimes B_{j_N}\right) = (K^{M-1}L^{N-1})\sum_{k=1}^{K}\sum_{l=1}^{L} S * (A_k\otimes B_l)\ . \tag{58}$$

Now, using the decomposition of C given by Eq. (53), and $S = |S\rangle\rangle\langle\langle S|$, one has that

$$\sum_a |C^{(a)}\rangle\rangle\langle\langle C^{(a)}| * \sum_{i_1,\ldots,i_M=1}^{K}\sum_{j_1,\ldots,j_N=1}^{L}\left(\bigotimes_{k=1}^{M} A_{i_k}\otimes\bigotimes_{l=1}^{N} B_{j_l}\right) = (K^{M-1}L^{N-1})|S\rangle\rangle\langle\langle S| * \sum_{k=1}^{K}\sum_{l=1}^{L}(A_k\otimes B_l)\ . \tag{59}$$

The positivity of each $|C^{(a)}\rangle\rangle\langle\langle C^{(a)}|$ implies that

$$|C^{(a)}\rangle\rangle\langle\langle C^{(a)}| * \sum_{i_1,\ldots,i_M=1}^{K}\sum_{j_1,\ldots,j_N=1}^{L}\left(\bigotimes_{k=1}^{M} A_{i_k}\otimes\bigotimes_{l=1}^{N} B_{j_l}\right) \le (K^{M-1}L^{N-1})|S\rangle\rangle\langle\langle S| * \sum_{k=1}^{K}\sum_{l=1}^{L}(A_k\otimes B_l) \tag{60}$$

for all a.

Consider now the case where $K = M, L = N$. Since A_i and B_j are Choi operators of unitary channels, they can be expressed as $A_i = |U_i\rangle\rangle\langle\langle U_i|$ and $B_j = |V_j\rangle\rangle\langle\langle V_j|$. Since the left-hand side of Eq. (60) is a sum of positive operators, the inequality also holds for the sum of any subset of the terms on the left-hand side. Then, by considering only the term in the sum over $i_1,\ldots,i_M, j_1,\ldots,j_N$ that corresponds to $i_k = k, j_l = l$ for all k, l, we obtain that

$$|C^{(a)}\rangle\rangle\langle\langle C^{(a)}| * \left(\bigotimes_{k=1}^{M}|U_k\rangle\rangle\langle\langle U_k|\otimes\bigotimes_{l=1}^{N}|V_l\rangle\rangle\langle\langle V_l|\right) \le (M^{(M-1)}N^{(N-1)})|S\rangle\rangle\langle\langle S| * \sum_{k=1}^{M}\sum_{l=1}^{N}\left(|U_k\rangle\rangle\langle\langle U_k|\otimes|V_l\rangle\rangle\langle\langle V_l|\right)\ , \tag{61}$$

for all a. Following the link product rule for vectors, as in, e.g., Lemma 1 of Ref. [10], the above equation can be rewritten as

$$\bigotimes_{k=1}^{M}\bigotimes_{l=1}^{N}(|C^{(a)}\rangle\rangle * |U_k\rangle\rangle\otimes|V_l\rangle\rangle)(\langle\langle C^{(a)}| * \langle\langle U_k|\otimes\langle\langle V_l|) \le (M^{(M-1)}N^{(N-1)})\sum_{k=1}^{M}\sum_{l=1}^{N}(|S\rangle\rangle * |U_k\rangle\rangle\otimes|V_l\rangle\rangle)(\langle\langle S| * \langle\langle U_k|\otimes\langle\langle V_l|)\ . \tag{62}$$

Now, invoking Lemma 1, we arrive at the conclusion that

$$|C^{(a)}\rangle\rangle * \bigotimes_{k=1}^{M}|U_k\rangle\rangle\otimes\bigotimes_{j=1}^{N}|V_j\rangle\rangle \in \mathrm{span}(\{|S\rangle\rangle * \left(|U_k\rangle\rangle\otimes|V_l\rangle\rangle\right)\}_{kl})\ , \tag{63}$$

for all a. Therefore, for every a, we have that

$$|C^{(a)}\rangle\rangle * \left(\bigotimes_{k=1}^{M}|U_k\rangle\rangle\otimes\bigotimes_{j=1}^{N}|V_j\rangle\rangle\right) = \sum_{k=1}^{M}\sum_{l=1}^{N}\xi_{kl}^{(a)}(\{U_i\}_i,\ \{V_j\}_j)\,|S\rangle\rangle * \left(|U_k\rangle\rangle\otimes|V_l\rangle\rangle\right)\ , \tag{64}$$

for some coefficients $\xi_{kl}^{(a)}(\{U_i\}_i,\ \{V_j\}_j) \in \mathbb{C}$ for all k, l.

□

3.3 Lemma 3 (for $M, N \in \mathbb{N}^+$)

Lemma 3. *Let $|S\rangle\rangle \in P\otimes I\otimes O\otimes I'\otimes O'\otimes F$, where I, O, I', O' correspond to d-dimensional Hilbert spaces and P, F correspond to $(2\times d)$-dimensional Hilbert spaces, and let $|C\rangle\rangle \in P\otimes I_1\otimes O_1\otimes\cdots\otimes I_M\otimes O_M\otimes I'_1\otimes O'_1\otimes\cdots\otimes I'_N\otimes O'_N\otimes F$, where $M, N \in \mathbb{N}^+$ and $\{I_i\}_i, \{O_i\}_i, \{I'_j\}_j, \{O'_j\}_j$ correspond to d-dimensional Hilbert spaces.*

If, for a given M, N, for all $(M+N)$-tuples of n-qubit unitary operators $(U_1, \ldots, U_M, V_1, \ldots, V_N)$,

$$|C\rangle\rangle * |U_1\rangle\rangle^{I_1 O_1} \otimes \cdots \otimes |U_M\rangle\rangle^{I_M O_M} \otimes |V_1\rangle\rangle^{I'_1 O'_1} \otimes \cdots \otimes |V_N\rangle\rangle^{I'_N O'_N} = \sum_{k=1}^{M} \sum_{l=1}^{N} \tilde{\xi}_{kl}(\{U_i\}_i,\ \{V_j\}_j) |S\rangle\rangle * |U_k\rangle\rangle^{I_k O_k} \otimes |V_l\rangle\rangle^{I'_l O'_l} \quad (65)$$

for some coefficients $\tilde{\xi}_{kl}(\{U_i\}_i,\ \{V_j\}_j) \in \mathbb{C}$ for all k, l that are simultaneously

1. *independent of U_k and V_l, and*
2. *linear in U_i and V_j for all $i \neq k, j \neq l$,*

then

$$|C\rangle\rangle^{P I_1 O_1 \ldots I_M O_M I'_1 O'_1 \ldots I'_N O'_N F} = \sum_{k=1}^{M} \sum_{l=1}^{N} |S\rangle\rangle^{P I_k O_k I'_l O'_l F} \otimes |\tilde{\xi}_{kl}\rangle\rangle^{\{I_1 O_1 \ldots I_M O_M I'_1 O'_1 \ldots I'_N O'_N\} \setminus \{I_k O_k I'_l O'_l\}} \quad (66)$$

for some vectors $|\tilde{\xi}_{kl}\rangle\rangle^{\{I_1 O_1 \ldots I_M O_M I'_1 O'_1 \ldots I'_N O'_N\} \setminus \{I_k O_k I'_l O'_l\}}$ that are independent of $\{U_i\}_i$ and $\{V_j\}_j$.

Proof. The first condition (independence) implies that we can write $\tilde{\xi}_{kl}(\{U_i\}_i,\ \{V_j\}_j) = \tilde{\xi}_{kl}(\{U_i\}_{i \neq k},\ \{V_j\}_{j \neq l})$. The second condition (linearity) combined with the Riesz representation theorem implies that we can write the linear functions $\tilde{\xi}_{kl}(\{U_i\}_{i \neq k},\ \{V_j\}_{j \neq l})$ using vectors $|\tilde{\xi}_{kl}\rangle\rangle$ according to

$$\tilde{\xi}_{kl}(\{U_i\}_{i \neq k},\ \{V_j\}_{j \neq l}) = |\tilde{\xi}_{kl}\rangle\rangle * \bigotimes_{i \neq k} |U_i\rangle\rangle \otimes \bigotimes_{j \neq l} |V_j\rangle\rangle\,, \quad (67)$$

where the definition of the link product of two vectors $|\psi\rangle, |\phi\rangle \in \mathbb{C}^d$ acting on the same space implies that $|\psi\rangle * |\phi\rangle = \overline{\langle\psi|}\, |\phi\rangle$, where the bar denotes complex conjugation.

Then, by explicitly writing in the system labels, for any sets of n-qubit unitaries $\{U_i\}_{i=1}^M$ and $\{V_j\}_{j=1}^N$, Eq. (65) becomes

$$\begin{aligned} &|C\rangle\rangle^{P I_1 O_1 \ldots I_M O_M I'_1 O'_1 \ldots I'_N O'_N F} * \left[\bigotimes_{i=1}^{M} |U_i\rangle\rangle^{I_i O_i} \otimes \bigotimes_{j=1}^{N} |V_j\rangle\rangle^{I'_j O'_j} \right] \\ &= \left[\sum_{k=1}^{M} \sum_{l=1}^{N} |\tilde{\xi}_{kl}\rangle\rangle^{\{I_1 O_1 \ldots I_M O_M I'_1 O'_1 \ldots I'_N O'_N\} \setminus \{I_k O_k I'_l O'_l\}} \otimes |S\rangle\rangle^{P I_k O_k I'_l O'_l F} \right] * \left[\bigotimes_{i=1}^{M} |U_i\rangle\rangle^{I_i O_i} \otimes \bigotimes_{j=1}^{N} |V_j\rangle\rangle^{I'_j O'_j} \right], \end{aligned} \quad (68)$$

for some vectors $|\tilde{\xi}_{kl}\rangle\rangle^{\{I_1 O_1 \ldots I_M O_M I'_1 O'_1 \ldots I'_N O'_N\} \setminus \{I_k O_k I'_l O'_l\}}$.

Since the equation is true for all unitaries $\{U_i\}$ and $\{V_j\}$, and the $\mathrm{span}(\{|U\rangle\rangle \,|\, U \in \mathrm{SU}(d)\}) = \mathbb{C}^d \otimes \mathbb{C}^d$, it implies that

$$|C\rangle\rangle^{P I_1 O_1 \ldots I_M O_M I'_1 O'_1 \ldots I'_N O'_N F} = \sum_{k=1}^{M} \sum_{l=1}^{N} |S\rangle\rangle^{P I_k O_k I'_l O'_l F} \otimes |\tilde{\xi}_{kl}\rangle\rangle^{\{I_1 O_1 \ldots I_M O_M I'_1 O'_1 \ldots I'_N O'_N\} \setminus \{I_k O_k I'_l O'_l\}} \quad (69)$$

for some vectors $|\tilde{\xi}_{kl}\rangle\rangle^{\{I_1 O_1 \ldots I_M O_M I'_1 O'_1 \ldots I'_N O'_N\} \setminus \{I_k O_k I'_l O'_l\}}$. □

3.4 Lemma 4 (for $M < 4^n/2 + 2$ and $N = 1$)

Lemma 4. *Let $|S\rangle\rangle \in P \otimes I \otimes O \otimes I' \otimes O' \otimes F$, where I, O, I', O' correspond to n-qubit Hilbert spaces for some $n \in \mathbb{N}^+$ and P, F correspond to $(n+1)$-qubit Hilbert spaces, and let $|C\rangle\rangle \in P \otimes I_1 \otimes O_1 \otimes \cdots \otimes I_M \otimes O_M \otimes I'_1 \otimes O'_1 \otimes F$, where $M \in \mathbb{N}^+$ and $\{I_i\}_i, \{O_i\}_i, I'_1, O'_1$ correspond to n-qubit Hilbert spaces.*

If, for all $(M+1)$-tuples of n-qubit unitary operators $(U_1,\dots,U_M,V_1)$, it holds that

$$|C\rangle\!\rangle * |U_1\rangle\!\rangle^{I_1O_1}\otimes\cdots\otimes|U_M\rangle\!\rangle^{I_MO_M}\otimes|V_1\rangle\!\rangle^{I_1'O_1'} = \sum_{k=1}^{M}\xi_{k1}|S\rangle\!\rangle * |U_k\rangle\!\rangle^{I_kO_k}\otimes|V_1\rangle\!\rangle^{I_1'O_1'} \tag{70}$$

for some complex numbers $\xi_{k1} \coloneqq \xi_{k1}(\{U_i\}_i,\ V_1)\in\mathbb{C}$, then there exist complex numbers $\tilde{\xi}_{k1} \coloneqq \tilde{\xi}_{k1}(\{U_i\}_i,\ V_1)\in\mathbb{C}$ such that Eq. (70) with $\{\xi_{k1}\}_{k=1}^M \leftarrow \{\tilde{\xi}_{k1}\}_{k=1}^M$ remains satisfied and, for all $k\in\{1,\dots,M\}$, $\tilde{\xi}_{k1}$ is simultaneously

1. *independent of U_k and V_1 (independence condition), and*

2. *linear in U_i for all $i\neq k$ (linearity condition),*

as long as $M < 4^n/2+2$.

Proof. Assume that Eq. (70) holds. Then, in particular, it holds for the choice $U_i=\sigma_{\vec{r}_i}$ for $i\in\{1,\dots,M\}$ and $V_1=\sigma_{\vec{q}_1}$, where $\vec{r}_i,\vec{q}_1\in\{0,1,2,3\}^{\times n}$ and $\{\sigma_{\vec{r}_i}\}_i$, $\sigma_{\vec{q}_1}$ are n-qubit Pauli operators. Here, the set of n-qubit Pauli operators is defined by

$$\left\{\sigma_{\vec{r}} \coloneqq \bigotimes_{i=1}^{n}\sigma_{r_i}\ \middle|\ \vec{r}\in\{0,1,2,3\}^{\times n}\right\}, \tag{71}$$

where $\sigma_0,\sigma_1,\sigma_2,\sigma_3$ are 1-qubit Pauli operators defined by

$$\sigma_0 \coloneqq \begin{pmatrix}1&0\\0&1\end{pmatrix},\quad \sigma_1 \coloneqq \begin{pmatrix}0&1\\1&0\end{pmatrix},\quad \sigma_2 \coloneqq \begin{pmatrix}0&-i\\i&0\end{pmatrix},\quad \sigma_3 \coloneqq \begin{pmatrix}1&0\\0&-1\end{pmatrix}. \tag{72}$$

Thus,

$$|C\rangle\!\rangle * |\sigma_{\vec{r}_1}\rangle\!\rangle^{I_1O_1}\otimes\cdots\otimes|\sigma_{\vec{r}_M}\rangle\!\rangle^{I_MO_M}\otimes|\sigma_{\vec{q}_1}\rangle\!\rangle^{I_1'O_1'} = \sum_{k=1}^{M}\xi_{k1}|S\rangle\!\rangle * |\sigma_{\vec{r}_k}\rangle\!\rangle^{I_kO_k}\otimes|\sigma_{\vec{q}_1}\rangle\!\rangle^{I_1'O_1'}. \tag{73}$$

Now suppose that $F\geq 1$ elements of the $\{\sigma_{\vec{r}_i}\}_{i=1}^M$ are equal to some fixed n-qubit Pauli operator $\sigma_{\vec{w}}$. Let the set of integers labeling those Pauli operators be denoted by $\mathbb{F}\coloneqq\{1\leq i\leq M|\sigma_{\vec{r}_i}=\sigma_{\vec{w}}\}$, such that $|\mathbb{F}|=F$. Equation (70) then reads

$$|C\rangle\!\rangle * \bigotimes_{i\in\mathbb{F}}|\sigma_{\vec{w}}\rangle\!\rangle^{I_iO_i}\otimes\bigotimes_{i\in\{1,\dots,M\}\setminus\mathbb{F}}|\sigma_{\vec{r}_i}\rangle\!\rangle^{I_iO_i}\otimes|\sigma_{\vec{q}_1}\rangle\!\rangle^{I_1'O_1'} = \sum_{k=1}^{M}\xi_{k1}^{\{\vec{r}_l\}_l,\vec{q}_1}|S\rangle\!\rangle * |\sigma_{\vec{r}_k}\rangle\!\rangle^{I_kO_k}\otimes|\sigma_{\vec{q}_1}\rangle\!\rangle^{I_1'O_1'}, \tag{74}$$

where, for the input unitaries chosen as n-qubit Paulis, we define

$$\xi_{k1}^{\{\vec{r}_l\}_l,\vec{q}_1} \coloneqq \xi_{k1}(\{U_i=\sigma_{\vec{r}_i}\}_{i=1}^M, V_1=\sigma_{\vec{q}_1})\ . \tag{75}$$

In the following, we will adopt the shorthand convention that any changes to the dependence of ξ_{k1} from $\xi_{k1}^{\{\vec{r}_l\}_l,\vec{q}_1}$ will be specified as $\xi_{k1}^{\{\vec{r}_l\}_l,\vec{q}_1}[U_i=(\dots),V_j=(\dots)]$, with all unspecified arguments U_i,V_1 defined to be the same as for $\xi_{k1}^{\{\vec{r}_l\}_l,\vec{q}_1}$ defined above. A key point that we note for later is that the value of each individual variable $\xi_{k1}^{\{\vec{r}_l\}_l,\vec{q}_1}$ for $k\in\mathbb{F}$ is not uniquely determined from Eq. (70), so we can take a different set of variables $\tilde{\xi}_{k1}$ still satisfying Eq. (70).

We now show that for all $k\in\{1,\dots,M\}$, the variables ξ_{k1} can be replaced by $\tilde{\xi}_{k1}$ defined by

$$\tilde{\xi}_{k1}(\{U_i=\sigma_{\vec{r}_i}\}_{i=1}^M, V_1=\sigma_{\vec{q}_1}) \coloneqq \xi_{k1}^{\{\vec{r}_l\}_l,\vec{q}_1}[U_k=\sigma_{\vec{r}_k^*}, V_1=\sigma_{\vec{q}^*}], \tag{76}$$

$$\tilde{\xi}_{k1}\left(\left\{U_i=\sum_{\vec{r}_i}\alpha_{\vec{r}_i}^i\sigma_{\vec{r}_i}\right\}_{i=1}^M,\ V_1=\sum_{\vec{q}_1}\beta_{\vec{q}_1}^1\sigma_{\vec{q}_1}\right) \coloneqq \sum_{\{\vec{r}_l\}_{l\neq k}}\left(\prod_{j\neq k}\alpha_{\vec{r}_j}^j\right)\tilde{\xi}_{k1}(\{U_i=\sigma_{\vec{r}_i}\}_{i=1}^M, V_1=\sigma_{\vec{q}_1}), \tag{77}$$

where $\vec{r}_k^* \in \{0,1,2,3\}^{\times n}$ is an arbitrary vector *outside* of the set $\{\vec{r}_1,\ldots,\vec{r}_{k-1},\vec{r}_{k+1},\ldots,\vec{r}_M\}$, $\vec{q}^* \in \{0,1,2,3\}^{\times n}$ is an arbitrary fixed vector, and $\alpha^i_{\vec{r}_i}, \beta^1_{\vec{q}_1}$ are complex numbers. In the discussion below, we pick one choice of $\vec{r}_k^*$ defined as a function of $\vec{r}_1,\ldots,\vec{r}_{k-1},\vec{r}_{k+1},\ldots,\vec{r}_M$, i.e., $\vec{r}_k^* = \vec{r}_k^*(\vec{r}_1,\ldots,\vec{r}_{k-1},\vec{r}_{k+1},\ldots,\vec{r}_M)$. Such an $\vec{r}_k^*$ always exists for $M < 4^n$.

By construction, if the definition in Eqs. (76)–(77) satisfies Eq. (70), then $\tilde{\xi}_{k1}$ satisfies both the linearity and independence conditions outlined in the statement of the lemma. We now proceed to show that the definition in Eqs. (76)–(77) indeed satisfies Eq. (70). We do this by considering the dependence of $\xi_{k1}^{\{\vec{r}_l\}_l,\vec{q}_1}$ on the unitaries $\{U_i = \sigma_{\vec{r}_i}\}_{i=1}^M$ and $V_1 = \sigma_{\vec{q}_1}$ in turn.

Dependence on $\{U_i\}_{i=1}^M$

We focus on the case where $\{U_i\}_i$, V_1 are chosen from the set of Pauli operators. For every $k \in \{1,\ldots M\}$, we choose one $\sigma_{\vec{r}_k^*}$ and take σ to be the unique n-qubit Pauli operator such that $\sigma\sigma_{\vec{r}_k} = \beta\sigma_{\vec{r}_k^*}$, where $\beta \in \{-1,1,i,-i\}$. We then consider the following expression

$$\mathbf{E} := \frac{\mathrm{d}}{\mathrm{d}\theta}\bigg|_{\theta=0}\left[|C\rangle\!\rangle * \bigotimes_{m\in\mathbb{F}_k} |e^{i\theta\sigma}\sigma_{\vec{r}_m}\rangle\!\rangle^{I_m O_m} * \bigotimes_{m=1|m\notin\mathbb{F}_k}^{M} |\sigma_{\vec{r}_m}\rangle\!\rangle^{I_m O_m} * |\sigma_{\vec{q}_1}\rangle\!\rangle^{I_1' O_1'} \right], \tag{78}$$

where $\mathbb{F}_k := \{1 \le i \le M \mid \sigma_{\vec{r}_i} = \sigma_{\vec{r}_k}\}$. Due to linearity, this expression can be evaluated in two ways: either by first computing the derivative and then applying Eq. (70), or by first applying Eq. (70) and then computing the derivative. The former method gives

$$\begin{aligned}
\mathbf{E} &= i\beta \sum_{m\in\mathbb{F}_k}\left[|C\rangle\!\rangle * |\sigma_{\vec{r}_k^*}\rangle\!\rangle^{I_m O_m} * \bigotimes_{i\in\mathbb{F}_k|i\neq m} |\sigma_{\vec{r}_i}\rangle\!\rangle^{I_i O_i} * \bigotimes_{i=1|i\notin\mathbb{F}_k}^{M} |\sigma_{\vec{r}_i}\rangle\!\rangle^{I_i O_i} * |\sigma_{\vec{q}_1}\rangle\!\rangle^{I_1' O_1'} \right] \\
&= i\beta \sum_{m\in\mathbb{F}_k}\Bigg[\xi_{m1}^{\{\vec{r}_l\}_l,\vec{q}_1}[U_m = \sigma_{\vec{r}_k^*}] |S\rangle\!\rangle * |\sigma_{\vec{r}_k^*}\rangle\!\rangle * |\sigma_{\vec{q}_1}\rangle\!\rangle \\
&\quad + \sum_{i\in\mathbb{F}_k|i\neq m} \xi_{i1}^{\{\vec{r}_l\}_l,\vec{q}_1}[U_m = \sigma_{\vec{r}_k^*}] |S\rangle\!\rangle * |\sigma_{\vec{r}_k}\rangle\!\rangle * |\sigma_{\vec{q}_1}\rangle\!\rangle \\
&\quad + \sum_{i=1|i\notin\mathbb{F}_k}^{M} \xi_{i1}^{\{\vec{r}_l\}_l,\vec{q}_1}[U_m = \sigma_{\vec{r}_k^*}] |S\rangle\!\rangle * |\sigma_{\vec{r}_i}\rangle\!\rangle * |\sigma_{\vec{q}_1}\rangle\!\rangle \Bigg],
\end{aligned} \tag{79}$$

while the latter gives

$$\begin{aligned}
\mathbf{E} &= \frac{\mathrm{d}}{\mathrm{d}\theta}\bigg|_{\theta=0}\Bigg[\sum_{m\in\mathbb{F}_k} \xi_{m1}^{\{\vec{r}_l\}_l,\vec{q}_1}[\{U_i = e^{i\theta\sigma}\sigma_{\vec{r}_k}\}_{i\in\mathbb{F}_k}] |S\rangle\!\rangle * |e^{i\theta\sigma}\sigma_{\vec{r}_k}\rangle\!\rangle^{I_m O_m} * |\sigma_{\vec{q}_1}\rangle\!\rangle^{I_1' O_1'} \\
&\quad + \sum_{m=1|m\notin\mathbb{F}_k}^{M} \xi_{m1}^{\{\vec{r}_l\}_l,\vec{q}_1}[\{U_i = e^{i\theta\sigma}\sigma_{\vec{r}_k}\}_{i\in\mathbb{F}_k}] |S\rangle\!\rangle * |\sigma_{\vec{r}_m}\rangle\!\rangle^{I_m O_m} * |\sigma_{\vec{q}_1}\rangle\!\rangle^{I_1' O_1'} \Bigg] \\
&= i\beta \sum_{m\in\mathbb{F}_k} \xi_{m1}^{\{\vec{r}_l\}_l,\vec{q}_1} |S\rangle\!\rangle * |\sigma_{\vec{r}_k^*}\rangle\!\rangle * |\sigma_{\vec{q}_l}\rangle\!\rangle \\
&\quad + \frac{\mathrm{d}}{\mathrm{d}\theta}\bigg|_{\theta=0}\left[\sum_{m\in\mathbb{F}_k} \xi_{m1}^{\{\vec{r}_l\}_l,\vec{q}_1}[\{U_i = e^{i\theta\sigma}\sigma_{\vec{r}_k}\}_{i\in\mathbb{F}_k}] \right] |S\rangle\!\rangle * |\sigma_{\vec{r}_k}\rangle\!\rangle * |\sigma_{\vec{q}_1}\rangle\!\rangle \\
&\quad + \sum_{\vec{v}\in\{\vec{r}_1,\ldots,\vec{r}_M\}\backslash\{\vec{r}_k\}} \frac{\mathrm{d}}{\mathrm{d}\theta}\bigg|_{\theta=0}\left[\sum_{m\in\mathbb{F}_{\vec{v}}} \xi_{m1}^{\{\vec{r}_l\}_l,\vec{q}_1}[\{U_i = e^{i\theta\sigma}\sigma_{\vec{r}_k}\}_{i\in\mathbb{F}_k}] \right] |S\rangle\!\rangle * |\sigma_{\vec{v}}\rangle\!\rangle * |\sigma_{\vec{q}_1}\rangle\!\rangle,
\end{aligned} \tag{80}$$

where $\mathbb{F}_{\vec{v}} := \{1 \leq m \leq M \mid \vec{r}_m = \vec{v}\}$. Note that the vector

$$|S\rangle\rangle * |\sigma_{\vec{r}_m}\rangle\rangle^{I_m O_m} * |\sigma_{\vec{q}_1}\rangle\rangle^{I_1' O_1'} \tag{81}$$

belongs to the Hilbert space $P \otimes F$, which is independent of I_m, O_m, I_1', O_1', thus the superscripts I_m, O_m, I_1', O_1' can be omitted. Also, note that

$$\sum_{m \in \mathbb{F}_k} \xi_{m1}^{\{\vec{r}_l\}_l, \vec{q}_1}[\{U_i = e^{i\theta\sigma}\sigma_{\vec{r}_k}\}_{i \in \mathbb{F}_k}] \tag{82}$$

and

$$\sum_{m \in \mathbb{F}_{\vec{v}}} \xi_{m1}^{\{\vec{r}_l\}_l, \vec{q}_1}[\{U_i = e^{i\theta\sigma}\sigma_{\vec{r}_k}\}_{i \in \mathbb{F}_k}] \tag{83}$$

are differentiable since their values are uniquely determined from

$$\begin{aligned}
&|C\rangle\rangle * \bigotimes_{m \in \mathbb{F}_k} |e^{i\theta\sigma}\sigma_{\vec{r}_m}\rangle\rangle^{I_m O_m} * \bigotimes_{m=1 | m \notin \mathbb{F}_k}^{M} |\sigma_{\vec{r}_m}\rangle\rangle^{I_m O_m} * |\sigma_{\vec{q}_1}\rangle\rangle^{I_1' O_1'} \\
&= \left(\sum_{m \in \mathbb{F}_k} \xi_{m1}^{\{\vec{r}_l\}_l, \vec{q}_1}[\{U_i = e^{i\theta\sigma}\sigma_{\vec{r}_k}\}_{i \in \mathbb{F}_k}] \right) |S\rangle\rangle * |e^{i\theta\sigma}\sigma_{\vec{r}_k}\rangle\rangle * |\sigma_{\vec{q}_1}\rangle\rangle \\
&+ \sum_{\vec{v} \in \{\vec{r}_1, \ldots, \vec{r}_M\} \setminus \{\vec{r}_k\}} \left(\sum_{m \in \mathbb{F}_{\vec{v}}} \xi_{m1}^{\{\vec{r}_l\}_l, \vec{q}_1}[\{U_i = e^{i\theta\sigma}\sigma_{\vec{r}_k}\}_{i \in \mathbb{F}_k}] \right) |S\rangle\rangle * |\sigma_{\vec{v}}\rangle\rangle * |\sigma_{\vec{q}_1}\rangle\rangle \,,
\end{aligned} \tag{84}$$

and thus can be obtained from the inner product of the vector on the left-hand side of Eq. (84) and vectors $|S\rangle\rangle * |e^{i\theta\sigma}\sigma_{\vec{r}_k}\rangle\rangle * |\sigma_{\vec{q}_1}\rangle\rangle$ or $|S\rangle\rangle * |\sigma_{\vec{v}}\rangle\rangle * |\sigma_{\vec{q}_1}\rangle\rangle$ (which are mutually orthogonal), which are polynomials of $e^{\pm i\theta}$.

By comparing the coefficients for $|S\rangle\rangle * |\sigma_{\vec{r}_k^*}\rangle\rangle * |\sigma_{\vec{q}_1}\rangle\rangle$, we find that

$$\sum_{m \in \mathbb{F}_k} \xi_{m1}^{\{\vec{r}_l\}_l, \vec{q}_1} = \sum_{m \in \mathbb{F}_k} \xi_{m1}^{\{\vec{r}_l\}_l, \vec{q}_1}[U_m = \sigma_{\vec{r}_k^*}]. \tag{85}$$

Dependence on V_1

In this part of the proof, we adopt the following shorthand notations. For any unitary operators U, V,

$$\begin{aligned}
|C[V]\rangle\rangle &:= |C\rangle\rangle * \bigotimes_{i=1}^{M} |\sigma_{\vec{r}_i}\rangle\rangle^{I_i O_i} * |V\rangle\rangle^{I_1' O_1'} \\
|S(U,V)\rangle\rangle &:= |S\rangle\rangle * |U\rangle\rangle * |V\rangle\rangle \\
b(\sigma_{\vec{v}}, V) &:= \sum_{m \in \mathbb{F}_{\vec{v}}} \xi_{m1}^{\{\vec{r}_l\}_l, \vec{q}_1}[V_1 = V] \,,
\end{aligned} \tag{86}$$

where $\mathbb{F}_{\vec{v}} := \{1 \leq i \leq M \mid \vec{r}_i = \vec{v}\}$.

For any two n-qubit Pauli operators σ_A, σ_B, either the operator $(\sigma_A + \sigma_B)/\sqrt{2}$ or the operator $(\sigma_A + i\sigma_B)/\sqrt{2}$ is

unitary. When $U := (\sigma_A + \beta\sigma_B)/\sqrt{2}$ with $\beta \in \{1, i\}$ is unitary, the following equality holds for any $\{\vec{r}_1, \ldots, \vec{r}_M\}, \sigma_A, \sigma_B$:

$$\begin{aligned} 0 =& |C[U]\rangle\rangle - \frac{1}{\sqrt{2}}(|C[\sigma_A]\rangle\rangle + \beta|C[\sigma_B]\rangle\rangle) \\ =& \frac{1}{\sqrt{2}} \sum_{\vec{v}\in\{\vec{r}_1,\ldots,\vec{r}_M\}} \left[\{b(\sigma_{\vec{v}}, U) - b(\sigma_{\vec{v}}, \sigma_A)\}|S(\sigma_{\vec{v}}, \sigma_A)\rangle\rangle + \beta\{b(\sigma_{\vec{v}}, U) - b(\sigma_{\vec{v}}, \sigma_B)\}|S(\sigma_{\vec{v}}, \sigma_B)\rangle\rangle\right]. \end{aligned} \tag{87}$$

We now calculate the inner product

$$\langle\langle S(\sigma_{\vec{v}}, \sigma_A)|S(\sigma_{\vec{v}'}, \sigma_B)\rangle\rangle = \mathrm{tr}(\sigma_B\sigma_A\sigma_{\vec{v}}\sigma_{\vec{v}'}) + \mathrm{tr}(\sigma_{\vec{v}}\sigma_A\sigma_B\sigma_{\vec{v}'}). \tag{88}$$

From this, it is clear that $\langle\langle S(\sigma_{\vec{v}}, \sigma_A)|S(\sigma_{\vec{v}'}, \sigma_B)\rangle\rangle = 0$ if $\sigma_{\vec{v}'} \not\propto \sigma_{\vec{v}}\sigma_A\sigma_B$. Therefore, taking an inner product of Eq. (87) with $|S(\sigma_{\vec{v}}, \sigma_A)\rangle\rangle$, we obtain

$$\begin{cases} \{b(\sigma_{\vec{v}}, U) - b(\sigma_{\vec{v}}, \sigma_A)\}\langle\langle S(\sigma_{\vec{v}}, \sigma_A)|S(\sigma_{\vec{v}}, \sigma_A)\rangle\rangle = 0 \quad \text{if for all } \ \sigma_{\vec{v}'} \in \{\sigma_{\vec{r}_1}, \ldots, \sigma_{\vec{r}_M}\} : \sigma_{\vec{v}'} \not\propto \sigma_{\vec{v}}\sigma_A\sigma_B \\ \\ \{b(\sigma_{\vec{v}}, U) - b(\sigma_{\vec{v}}, \sigma_A)\}\langle\langle S(\sigma_{\vec{v}}, \sigma_A)|S(\sigma_{\vec{v}}, \sigma_A)\rangle\rangle \\ \quad +\beta\{b(\gamma\sigma_{\vec{v}}\sigma_A\sigma_B, U) - b(\gamma\sigma_{\vec{v}}\sigma_A\sigma_B, \sigma_B)\}\langle\langle S(\sigma_{\vec{v}}, \sigma_A)|S(\gamma\sigma_{\vec{v}}\sigma_A\sigma_B, \sigma_B)\rangle\rangle = 0 \quad \text{else,} \end{cases} \tag{89}$$

where $\gamma \in \{1, -1, i, -i\}$ is defined by the unique choice of $\sigma_{\vec{v}'} \in \{\sigma_{\vec{r}_1}, \ldots, \sigma_{\vec{r}_M}\}$ such that

$$\sigma_{\vec{v}'} = \gamma\sigma_{\vec{v}}\sigma_A\sigma_B. \tag{90}$$

In the first case, i.e., if for all $\sigma_{\vec{v}'} \in \{\sigma_{\vec{r}_1}, \ldots, \sigma_{\vec{r}_M}\} : \sigma_{\vec{v}'} \not\propto \sigma_{\vec{v}}\sigma_A\sigma_B$, we directly obtain

$$b(\sigma_{\vec{v}}, U) - b(\sigma_{\vec{v}}, \sigma_A) = 0. \tag{91}$$

In the second case, if

$$\langle\langle S(\sigma_{\vec{v}}, \sigma_A)|S(\gamma\sigma_{\vec{v}}\sigma_A\sigma_B, \sigma_B)\rangle\rangle = \mathrm{tr}(\sigma_B\sigma_A\sigma_{\vec{v}}\gamma\sigma_{\vec{v}}\sigma_A\sigma_B) + \mathrm{tr}(\sigma_{\vec{v}}\sigma_A\sigma_B\gamma\sigma_{\vec{v}}\sigma_A\sigma_B) = 0 \tag{92}$$

holds, we also obtain Eq. (91)

By a similar argument, if for all $\sigma_{\vec{v}''} \in \{\sigma_{\vec{r}_1}, \ldots, \sigma_{\vec{r}_M}\} : \sigma_{\vec{v}''} \not\propto \sigma_{\vec{v}}\sigma_B\sigma_A$, we directly obtain

$$b(\sigma_{\vec{v}}, U) - b(\sigma_{\vec{v}}, \sigma_B) = 0. \tag{93}$$

Alternatively, if

$$\langle\langle S(\sigma_{\vec{v}}, \sigma_B)|S(\gamma\sigma_{\vec{v}}\sigma_B\sigma_A, \sigma_A)\rangle\rangle = \mathrm{tr}(\sigma_A\sigma_B\sigma_{\vec{v}}\delta\sigma_{\vec{v}}\sigma_B\sigma_A) + \mathrm{tr}(\sigma_{\vec{v}}\sigma_B\sigma_A\delta\sigma_{\vec{v}}\sigma_B\sigma_A) = 0 \tag{94}$$

holds, where $\delta \in \{1, -1, i, -i\}$ is defined by the unique choice of $\sigma_{\vec{v}''} \in \{\sigma_{\vec{r}_1}, \ldots, \sigma_{\vec{r}_M}\}$ such that

$$\sigma_{\vec{v}''} = \delta\sigma_{\vec{v}}\sigma_B\sigma_A, \tag{95}$$

we also obtain Eq. (93).

Consider now the conditions for Eq. (92) to be satisfied. The first term is given by $\mathrm{tr}(\sigma_B\sigma_A\sigma_{\vec{v}}\gamma\sigma_{\vec{v}}\sigma_A\sigma_B) = \gamma\mathrm{tr}\mathbb{1}$. For the second term, there are four cases:

- If $\gamma = \pm 1$, then $\pm\sigma_{\vec{v}}\sigma_A\sigma_B$ is an n-qubit Pauli operator so the second term is given by $\mathrm{tr}[\sigma_{\vec{v}}\sigma_A\sigma_B\gamma\sigma_{\vec{v}}\sigma_A\sigma_B] = \gamma\mathrm{tr}[(\pm\sigma_{\vec{v}}\sigma_A\sigma_B)(\pm\sigma_{\vec{v}}\sigma_A\sigma_B)] = \gamma\mathrm{tr}\mathbb{1}$.

- If $\gamma = \pm i$, then $\pm i\sigma_{\vec{v}}\sigma_A\sigma_B$ is an n-qubit Pauli operator so the second term is given by $\mathrm{tr}[(\sigma_{\vec{v}}\sigma_A\sigma_B)\gamma(\sigma_{\vec{v}}\sigma_A\sigma_B)] = -\gamma\mathrm{tr}[(\pm i\sigma_{\vec{v}}\sigma_A\sigma_B)(\pm i\sigma_{\vec{v}}\sigma_A\sigma_B)] = -\gamma\mathrm{tr}\mathbb{1}$.

Therefore, Eq. (92) is satisfied if and only if $\gamma = \pm i$. By a similar argument, Eq. (94) is satisfied if and only if $\delta = \pm i$.

Note that the following equivalences hold: $\forall \sigma_{\vec{v}''} \in \{\sigma_{\vec{r}_1}, \ldots, \sigma_{\vec{r}_M}\} : \sigma_{\vec{v}''} \not\propto \sigma_{\vec{v}}\sigma_B\sigma_A \iff \forall \sigma_{\vec{v}'} \in \{\sigma_{\vec{r}_1}, \ldots, \sigma_{\vec{r}_M}\} : \sigma_{\vec{v}'} \not\propto \sigma_{\vec{v}}\sigma_A\sigma_B$, and also $\gamma \in \{i, -i\} \iff \delta \in \{i, -i\}$. Therefore, for all $\{\sigma_{\vec{r}_1}, \ldots, \sigma_{\vec{r}_M}\}$, for every tuple $(\sigma_{\vec{v}} \in \{\sigma_{\vec{r}_1}, \ldots, \sigma_{\vec{r}_M}\}, \sigma_A, \sigma_B)$, if one of the two following conditions is satisfied:

1. $\forall\, \sigma_{\vec{v}'} \in \{\sigma_{\vec{r}_1}, \ldots, \sigma_{\vec{r}_M}\} : \sigma_{\vec{v}'} \not\propto \sigma_{\vec{v}}\sigma_A\sigma_B$, or

2. $\exists\, \sigma_{\vec{v}'}$ such that $\sigma_{\vec{v}'} = \pm i\sigma_{\vec{v}}\sigma_A\sigma_B$,

then

$$b(\sigma_{\vec{v}}, \sigma_A) = b(\sigma_{\vec{v}}, U)\,, \tag{96}$$
$$b(\sigma_{\vec{v}}, \sigma_B) = b(\sigma_{\vec{v}}, U)\,, \tag{97}$$

which implies that

$$b(\sigma_{\vec{v}}, \sigma_A) = b(\sigma_{\vec{v}}, \sigma_B)\,. \tag{98}$$

We now consider the choices of $(\sigma_{\vec{v}}, \sigma_A, \sigma_B)$ where neither of the above two conditions is satisfied.

The case where $\sigma_{\vec{v}} = \sigma_A$ or $\sigma_{\vec{v}} = \sigma_B$: First, note that if $\sigma_{\vec{v}} = \sigma_B$, and Condition 1. is not satisfied, then Eq. (90) implies that $\sigma_{\vec{v}'} = \sigma_A$ and $\gamma = \pm 1$, so Condition 2. is not satisfied either. In this case, we can take another n-qubit Pauli operator $\sigma_C \notin \{\sigma_{\vec{r}_1}, \ldots, \sigma_{\vec{r}_M}\}$ (which implies that $\sigma_C \neq \sigma_A, \sigma_B$), such that $\sigma_B\sigma_A\sigma_C = \pm i\sigma_{BAC}$ for some n-qubit Pauli σ_{BAC}. The existence of such a σ_C is guaranteed by:

- the fact that half of the total 4^n number of n-qubit Pauli operators, when multiplied after $\sigma_B\sigma_A$ (which is not equal to the identity because $\sigma_B \neq \sigma_A$ by construction), gives a Pauli operator times ± 1, and the other half will give a Pauli operator times $\pm i$,

- the fact that the set $\{\sigma_{\vec{r}_1}, \ldots, \sigma_{\vec{r}_M}\}$ contains the operators σ_A, σ_B, which, when multiplied after $\sigma_B\sigma_A$, gives a Pauli operator times ± 1,

- the assumption that $M < 4^n/2 + 2$.

Applying the procedure in Eqs. (87)–(98) above to the unitary $U' := (\sigma_C + \beta'\sigma_B)/\sqrt{2}$ (with $\beta' \in \{1, i\}$), we find that there is no $\sigma_{\vec{v}'} \in \{\sigma_{\vec{r}_1}, \ldots, \sigma_{\vec{r}_M}\}$ such that $\sigma_{\vec{v}'} \propto \sigma_{\vec{v}}\sigma_C\sigma_B = \sigma_B\sigma_C\sigma_B \propto \sigma_C$. Therefore, Condition 1. is satisfied for $(\sigma_{\vec{v}} \in \{\sigma_{\vec{r}_1}, \ldots, \sigma_{\vec{r}_M}\}, \sigma_C, \sigma_B)$ and we have that

$$b(\sigma_{\vec{v}}, \sigma_C) = b(\sigma_{\vec{v}}, U') = b(\sigma_{\vec{v}}, \sigma_B)\,. \tag{99}$$

Applying the procedure in Eqs. (87)–(98) above to the unitary $U'' := (\sigma_A + \beta''\sigma_C)/\sqrt{2}$ (with $\beta'' \in \{1, i\}$), we find that either (a) there is no $\sigma_{\vec{v}''} \in \{\sigma_{\vec{r}_1}, \ldots, \sigma_{\vec{r}_M}\}$ such that $\sigma_{\vec{v}''} \propto \sigma_{\vec{v}}\sigma_A\sigma_C$, or (b) if there is, then $\sigma_{\vec{v}}\sigma_A\sigma_C = \sigma_B\sigma_A\sigma_C = \pm i\sigma_{BAC}$, i.e. $\sigma_{\vec{v}''} = \sigma_{BAC}$. Therefore, for $(\sigma_{\vec{v}} \in \{\sigma_{\vec{r}_1}, \ldots, \sigma_{\vec{r}_M}\}, \sigma_A, \sigma_C)$, either Condition 1. or 2. is satisfied and we have that

$$b(\sigma_{\vec{v}}, \sigma_A) = b(\sigma_{\vec{v}}, U'') = b(\sigma_{\vec{v}}, \sigma_C)\,. \tag{100}$$

Overall, Eq. (98) is satisfied for $(\sigma_{\vec{v}} \in \{\sigma_{\vec{r}_1}, \ldots, \sigma_{\vec{r}_M}\}, \sigma_A, \sigma_B)$. An analogous argument applies if $\sigma_{\vec{v}} = \sigma_A$.

The case where $\sigma_{\vec{v}} \neq \sigma_A, \sigma_B$: If neither Condition 1. nor Condition 2. are satisfied, then we can take another n-qubit Pauli operator $\sigma_C \neq \sigma_A, \sigma_B$, such that

$$\sigma_{\vec{v}}\sigma_A\sigma_C = \pm i\sigma_{vAC}\,, \tag{101}$$
$$\sigma_{\vec{v}}\sigma_C\sigma_B \in \{\pm i\sigma_{vCB}\} \iff \sigma_{\vec{v}}\sigma_B\sigma_C \in \{\pm i\sigma_{vCB}\}\,, \tag{102}$$

for some n-qubit Pauli operators $\sigma_{vAC}, \sigma_{vCB}$. The existence of such a σ_C is guaranteed by:

- the fact that for any two different non-identity n-qubit Pauli operators σ_E, σ_F, there exists an n-qubit Pauli operator σ_Q such that both $\sigma_E\sigma_Q$ and $\sigma_F\sigma_Q$ are equal to $+i$ or $-i$ times an n-qubit Pauli operator,

- the fact that for any two different non-identity n-qubit Pauli operators σ_E, σ_F, there exists an n-qubit Pauli operator σ_R such that $\sigma_E\sigma_R$ equals $\pm i$ times an n-qubit Pauli operator, while $\sigma_F\sigma_R$ equals ± 1 times an n-qubit Pauli operator.

This enables σ_C to be chosen according to the following strategy:

- if there are σ_E, σ_F such that $\sigma_E = \pm\sigma_v\sigma_A$ and $\sigma_F = \pm\sigma_v\sigma_B$, then pick $\sigma_C = \sigma_Q$ as defined above,
- if there are σ_E, σ_F such that either $\sigma_E = \pm\sigma_v\sigma_A$ and $\sigma_F = \pm i\sigma_v\sigma_B$, or $\sigma_F = \pm i\sigma_v\sigma_A$ and $\sigma_E = \pm\sigma_v\sigma_B$, then pick $\sigma_C = \sigma_R$ as defined above,
- if there are σ_E, σ_F such that $\sigma_E = \pm i\sigma_v\sigma_A$ and $\sigma_F = \pm i\sigma_v\sigma_B$, then pick $\sigma_C = \sigma_F$, in which case $\sigma_{\vec{v}}\sigma_B\sigma_C = \sigma_{\vec{v}}\sigma_B(\pm i\sigma_v\sigma_B) = \mp i\mathbb{1}$ and $\sigma_{\vec{v}}\sigma_A\sigma_C = \pm i\sigma_{\vec{v}}\sigma_A\sigma_{\vec{v}}\sigma_B = \mp i\sigma_{\vec{v}}\sigma_{\vec{v}}\sigma_A\sigma_B = \mp i\sigma_{\vec{v}}\sigma_B\sigma_A\sigma_{\vec{v}} = -\sigma_C\sigma_A\sigma_{\vec{v}} = -(\sigma_{\vec{v}}\sigma_A\sigma_C)^\dagger$ (where in the third equality we use the assumption that Conditions 1. and 2. are not satisfied, which implies that $\sigma_{\vec{v}}\sigma_A\sigma_B = \sigma_B\sigma_A\sigma_{\vec{v}}$), and therefore $\sigma_{\vec{v}}\sigma_A\sigma_C$ must be proportional to $\pm i$ times a Pauli.

Applying the procedure in Eqs. (87)–(98) above to the unitary $U' := (\sigma_C + \beta'\sigma_B)/\sqrt{2}$ (with $\beta' \in \{1, i\}$), we find that Condition 1. or 2. is satisfied for $(\sigma_{\vec{v}} \in \{\sigma_{\vec{r}_1}, \ldots, \sigma_{\vec{r}_M}\}, \sigma_C, \sigma_B)$ and we have that

$$b(\sigma_{\vec{v}}, \sigma_C) = b(\sigma_{\vec{v}}, U') = b(\sigma_{\vec{v}}, \sigma_B)\,. \tag{103}$$

Applying the procedure in Eqs. (87)–(98) above to the unitary $U'' := (\sigma_A + \beta''\sigma_C)/\sqrt{2}$ (with $\beta'' \in \{1, i\}$), we find that Condition 1. or 2. is satisfied for $(\sigma_{\vec{v}} \in \{\sigma_{\vec{r}_1}, \ldots, \sigma_{\vec{r}_M}\}, \sigma_A, \sigma_C)$ and we have that

$$b(\sigma_{\vec{v}}, \sigma_A) = b(\sigma_{\vec{v}}, U'') = b(\sigma_{\vec{v}}, \sigma_C)\,. \tag{104}$$

Overall, Eq. (98) is satisfied for $(\sigma_{\vec{v}} \in \{\sigma_{\vec{r}_1}, \ldots, \sigma_{\vec{r}_M}\}, \sigma_A, \sigma_B)$.

Having shown that for all $\{\sigma_{\vec{r}_1}, \ldots, \sigma_{\vec{r}_M}\}$, for every tuple $(\sigma_{\vec{v}} \in \{\sigma_{\vec{r}_1}, \ldots, \sigma_{\vec{r}_M}\}, \sigma_A, \sigma_B)$, Eq. (98) is satisfied, we conclude that $b(\sigma_{\vec{v}}, \sigma_{\vec{q}_1}) := \sum_{m\in\mathbb{F}_{\vec{v}}} \xi_{m1}^{\{\vec{r}_l\}_l, \vec{q}_1}$ is independent of the choice of $\sigma_{\vec{q}_1}$. This means that

$$\sum_{m\in\mathbb{F}_{\vec{v}}} \xi_{m1}^{\{\vec{r}_l\}_l, \vec{q}_1} = \sum_{m\in\mathbb{F}_{\vec{v}}} \xi_{m1}^{\{\vec{r}_l\}_l, \vec{q}_1}[V_1 = \sigma_{\vec{q}^*}]\,, \tag{105}$$

for any n-qubit Pauli operator $\sigma_{\vec{q}^*}$.

Proving that the redefinition of Eqs. (76) and (77) satisfies Eq. (70)

Equations (85) and Eq. (105) together show that for all $\vec{r}_1, \ldots, \vec{r}_M, \vec{q}_1 \in \{0, 1, 2, 3\}^{\times n}$,

$$\begin{aligned}
|C\rangle\!\rangle * \bigotimes_{i=1}^{M} |\sigma_{\vec{r}_i}\rangle\!\rangle^{I_iO_i} * |\sigma_{\vec{q}_1}\rangle\!\rangle^{I_1'O_1'} &= \sum_{k=1}^{M} \xi_{k1}^{\{\vec{r}_l\}_l, \vec{q}_1} |S\rangle\!\rangle * |\sigma_{\vec{r}_k}\rangle\!\rangle * |\sigma_{\vec{q}_1}\rangle\!\rangle \\
&= \sum_{\vec{v}\in\{\vec{r}_1,\ldots,\vec{r}_M\}} \sum_{m\in\mathbb{F}_{\vec{v}}} \xi_{m1}^{\{\vec{r}_l\}_l, \vec{q}_1} |S\rangle\!\rangle * |\sigma_{\vec{v}}\rangle\!\rangle * |\sigma_{\vec{q}_1}\rangle\!\rangle \\
&= \sum_{\vec{v}\in\{\vec{r}_1,\ldots,\vec{r}_M\}} \sum_{m\in\mathbb{F}_{\vec{v}}} \xi_{m1}^{\{\vec{r}_l\}_l, \vec{q}_1}[V_1 = \sigma_{\vec{q}^*}] |S\rangle\!\rangle * |\sigma_{\vec{v}}\rangle\!\rangle * |\sigma_{\vec{q}_1}\rangle\!\rangle \\
&= \sum_{\vec{v}\in\{\vec{r}_1,\ldots,\vec{r}_M\}} \sum_{m\in\mathbb{F}_{\vec{v}}} \xi_{m1}^{\{\vec{r}_l\}_l, \vec{q}_1}[U_m = \sigma_{\vec{v}^*}, V_1 = \sigma_{\vec{q}^*}] |S\rangle\!\rangle * |\sigma_{\vec{v}}\rangle\!\rangle * |\sigma_{\vec{q}_1}\rangle\!\rangle \\
&= \sum_{k=1}^{M} \tilde{\xi}_{k1}(\{U_i = \sigma_{\vec{r}_i}\}_{i=1}^{M}, V_1 = \sigma_{\vec{q}_1}) |S\rangle\!\rangle * |\sigma_{\vec{r}_k}\rangle\!\rangle * |\sigma_{\vec{q}_1}\rangle\!\rangle\,,
\end{aligned} \tag{106}$$

where $\mathbb{F}_{\vec{v}} := \{1 \le i \le M \mid \vec{r}_i = \vec{v}\}$, $\vec{v}^* \in \{0, 1, 2, 3\}^{\times n}$ is an arbitrary vector outside of the set $\{\vec{r}_1, \ldots, \vec{r}_M\}\backslash\{\vec{v}\}$, and $\vec{q}^* \in \{0, 1, 2, 3\}^{\times n}$ is an arbitrary fixed vector. Therefore, $\tilde{\xi}_{k1}$ as defined in Eq. (76) indeed satisfies Eq. (70).

This also implies that

$$
\begin{aligned}
&|C\rangle\!\rangle * \bigotimes_{i=1}^{M} |\sum_{\vec{r}_i} \alpha^i_{\vec{r}_i}\sigma_{\vec{r}_i}\rangle\!\rangle^{I_iO_i} * |\sum_{\vec{q}_1} \beta^1_{\vec{q}_1}\sigma_{\vec{q}_1}\rangle\!\rangle^{I'_1O'_1} = \sum_{\{\vec{r}_l\}_l,\vec{q}_1} \left(\prod_{j=1}^{M} \alpha^j_{\vec{r}_j}\right) \beta^1_{\vec{q}_1} |C\rangle\!\rangle * \bigotimes_{i=1}^{M} |\sigma_{\vec{r}_i}\rangle\!\rangle^{I_iO_i} * |\sigma_{\vec{q}_1}\rangle\!\rangle^{I'_1O'_1} \\
&= \sum_{\{\vec{r}_l\}_l,\vec{q}_1} \left(\prod_{j=1}^{M} \alpha^j_{\vec{r}_j}\right) \beta^1_{\vec{q}_1} \left[\sum_{k=1}^{M} \tilde{\xi}_{k1}(\{U_m = \sigma_{\vec{r}_m}\}_m, V_1 = \sigma_{\vec{q}_1}) |S\rangle\!\rangle * |\sigma_{\vec{r}_k}\rangle\!\rangle^{I_kO_k} * |\sigma_{\vec{q}_1}\rangle\!\rangle^{I'_1O'_1}\right] \\
&= \sum_{k=1}^{M} \left[\sum_{\{\vec{r}_l\}_{l\neq k}} \left(\prod_{j=1|j\neq k}^{M} \alpha^j_{\vec{r}_j}\right) \tilde{\xi}_{k1}(\{U_m = \sigma_{\vec{r}_m}\}_m, V_1 = \sigma_{\vec{q}_1})\right] \sum_{\vec{r}_k,\vec{q}_1} \alpha^k_{\vec{r}_k}\beta^1_{\vec{q}_1} |S\rangle\!\rangle * |\sigma_{\vec{r}_k}\rangle\!\rangle^{I_kO_k} * |\sigma_{\vec{q}_1}\rangle\!\rangle^{I'_1O'_1} \\
&= \sum_{k=1}^{M} \tilde{\xi}_{k1} \left(\left\{U_i = \sum_{\vec{r}_i} \alpha^i_{\vec{r}_i}\sigma_{\vec{r}_i}\right\},\ V_1 = \sum_{\vec{q}_1} \beta^1_{\vec{q}_1}\sigma_{\vec{q}_1}\right) |S\rangle\!\rangle * |\sum_{\vec{r}_k} \alpha^k_{\vec{r}_k}\sigma_{\vec{r}_k}\rangle\!\rangle^{I_kO_k} * |\sum_{\vec{q}_1} \beta^1_{\vec{q}_1}\sigma_{\vec{q}_1}\rangle\!\rangle^{I'_1O'_1}, \qquad (107)
\end{aligned}
$$

i.e., Eq. (77) also satisfies Eq. (70). □

3.5 Lemma 5 (for $\max\{M, N\} \leq \max\{2, d-1\}$)

Before proving Lemma 5, we present the definition of a QC-CC higher-order transformation with an arbitrary number of slots $M+N$ [3].

An operator $C \in \mathcal{L}(P \otimes I_1 \otimes O_1 \otimes \ldots \otimes I_{M+N} \otimes O_{M+N} \otimes F)$ corresponds to the Choi operator of a $(M+N)$-slot QC-CC higher-order transformation if it satisfies

$$
\begin{aligned}
&C = \sum_{\vec{r}_{M+N} \in \mathrm{Perm}(1,\ldots,M+N)} C_{P\vec{r}_{M+N}F}, && (108)\\
&\text{such that} \qquad C_{P\vec{r}_{M+N}F} \geq 0 \quad \forall\, \vec{r}_{M+N}, && (109)\\
&\mathrm{tr}_F[C_{P\vec{r}_{M+N}F}] = C_{P\vec{r}_{M+N}} \otimes \mathbb{1}^{O_{r_{M+N}}} \quad \forall\, \vec{r}_{M+N}, && (110)\\
&\sum_{r_{m+1}} \mathrm{tr}_{I_{r_{m+1}}}[C_{P\vec{r}_m r_{m+1}}] = C_{P\vec{r}_m} \otimes \mathbb{1}^{O_{r_m}} \quad \forall\, m \in \{1,\ldots,M+N-1\}, \forall\, \vec{r}_m \coloneqq (r_1,\ldots,r_m), && (111)\\
&\sum_{r_1} \mathrm{tr}_{I_{r_1}}[C_{Pr_1}] = \mathbb{1}^P, && (112)
\end{aligned}
$$

where $\vec{r}_m r_{m+1}$ represents a vector $(r_1, \ldots, r_m, r_{m+1})$, with each vector $\vec{r}_m$ composed of elements $r_1, \ldots, r_m$, and the operators $C_{P\vec{r}_m} \in \mathcal{L}(P \otimes I_1 \otimes O_1 \otimes \cdots \otimes I_{m-1} \otimes O_{m-1} \otimes I_m)$ for $m \in \{1, \ldots, M+N\}$ are recursively defined by

$$
\begin{aligned}
C_{P\vec{r}_{M+N}} &\coloneqq \frac{1}{d}\mathrm{tr}_{O_{r_{M+N}}F}[C_{P\vec{r}_{M+N}F}], && (113)\\
C_{P\vec{r}_m} &\coloneqq \frac{1}{d}\sum_{r_{m+1}} \mathrm{tr}_{O_{r_m}I_{r_{m+1}}}[C_{P\vec{r}_m r_{m+1}}] \quad \forall\, m \in \{1,\ldots,M+N-1\}. && (114)
\end{aligned}
$$

These conditions will be referred as the *QC-CC conditions*. We remark that quantum combs are a subset of QC-CCs.

For convenience in the following, we rename the last N input and output systems as

$$
I'_k \coloneqq I_{M+k}, \quad O'_k \coloneqq O_{M+k} \quad \forall\, k \in \{1,\ldots,N\}. \qquad (115)
$$

Lemma 5. *Let $|S\rangle\!\rangle \in P \otimes I \otimes O \otimes I' \otimes O' \otimes F$, where I, O, I', O' correspond to d-dimensional Hilbert spaces and P, F correspond to $(2 \times d)$-dimensional Hilbert spaces, be the Choi vector of the quantum switch and let $C \in \mathcal{L}(P \otimes I_1 \otimes$*

$O_1 \otimes \ldots \otimes I_M \otimes O_M \otimes I'_1 \otimes O'_1 \otimes \ldots \otimes I'_N \otimes O'_N \otimes F$), where $M, N \in \mathbb{N}^+$ and $\{I_i\}_i, \{O_i\}_i, \{I'_j\}_j, \{O'_j\}_j$ correspond to d-dimensional Hilbert spaces, be the Choi operator of an $(M+N)$-slot QC-CC higher-order transformation, which we write as

$$C = \sum_{\vec{r}_{M+N}} C_{P\vec{r}_{M+N}F}\,, \tag{116}$$

with $\vec{r}_{M+N} \in \mathrm{Perm}(1, \ldots, M+N)$, where all $C_{P\vec{r}_{M+N}F} \geq 0$ and, hence, can be decomposed as

$$C_{P\vec{r}_{M+N}F} = \sum_a |C^{(a)}_{P\vec{r}_{M+N}F}\rangle\rangle\langle\langle C^{(a)}_{P\vec{r}_{M+N}F}|\,. \tag{117}$$

If $\max(M, N) \leq \max(2, d-1)$, then the set of operators $|C^{(a)}_{P\vec{r}_{M+N}F}\rangle\rangle\langle\langle C^{(a)}_{P\vec{r}_{M+N}F}|$ cannot be such that

$$|C^{(a)}_{P\vec{r}_{M+N}F}\rangle\rangle = \sum_{i=1}^{M}\sum_{k=1}^{N} |S\rangle\rangle^{PI_iO_iI'_kO'_kF} \otimes |\tilde{\xi}^{(a),\vec{r}_{M+N}}_{ik}\rangle\rangle, \tag{118}$$

for all $\vec{r}_{M+N}$ and a, where $|\tilde{\xi}^{(a),\vec{r}_{M+N}}_{ik}\rangle\rangle \in I_{\bar{i}} \otimes O_{\bar{i}} \otimes I'_{\bar{k}} \otimes O'_{\bar{k}}$ for all $i \in \{1, \ldots, M\}$ and $k \in \{1, \ldots, N\}$, and $I_{\bar{i}} \coloneqq \bigotimes_{i' \neq i} I_{i'}$, $O_{\bar{i}} \coloneqq \bigotimes_{i' \neq i} O_{i'}$, $I'_{\bar{k}} \coloneqq \bigotimes_{k' \neq k} I'_{k'}$, and $O'_{\bar{k}} \coloneqq \bigotimes_{k' \neq k} O'_{k'}$.

Proof. The proof follows by contradiction. To this end, we use Eqs. (110) and (111) in the QC-CC conditions to show the following equation for $C_{P\vec{r}_m}$:

$$\sum_{r_m} \mathrm{tr}_{I_{r_m}}[C_{P\vec{r}_m}] = \sum_{i,j \in \mathbb{A}_{\vec{r}_{m-1}}} \sum_{k,l \in \mathbb{B}_{\vec{r}_{m-1}}} \Big(|0\rangle\langle 0|^{P_C} \otimes |\mathbb{1}\rangle\rangle^{P_T I_i} \langle\langle \mathbb{1}|^{P_T I_j} \otimes |\mathbb{1}\rangle\rangle^{O_i I'_k} \langle\langle \mathbb{1}|^{O_j I'_l} \otimes \mathbb{1}^{O'_l \to O'_k}$$
$$+ |1\rangle\langle 1|^{P_C} \otimes |\mathbb{1}\rangle\rangle^{P_T I'_k} \langle\langle \mathbb{1}|^{P_T I'_l} \otimes |\mathbb{1}\rangle\rangle^{O'_k I_i} \langle\langle \mathbb{1}|^{O'_l I_j} \otimes \mathbb{1}^{O_j \to O_i} \Big) \otimes C^{(ijkl)}_{P\vec{r}_{m-1}} \quad \forall m \in \{1, \ldots, M+N+1\}, \tag{119}$$

where the summation over r_m for $m = M+N+1$ is taken as $I_{r_{M+N+1}} \coloneqq F$, $C_{P\vec{r}_{M+N+1}}$ is defined by $C_{P\vec{r}_{M+N+1}} \coloneqq C_{P\vec{r}_{M+N}F}$, the set of indices $\mathbb{A}_{\vec{r}_{m-1}}$ and $\mathbb{B}_{\vec{r}_{m-1}}$ are defined by

$$\mathbb{A}_{\vec{r}_{m-1}} \coloneqq \{r_1, \ldots, r_{m-1}\} \cap \{1, \ldots, M\}, \tag{120}$$
$$\mathbb{B}_{\vec{r}_{m-1}} \coloneqq \{r_1 - M, \ldots, r_{m-1} - M\} \cap \{1, \ldots, N\}, \tag{121}$$

and $C^{(ijkl)}_{P\vec{r}_{m-1}}$ is an operator. If this equation holds, since $\mathbb{A}_{\vec{r}_0}$ and $\mathbb{B}_{\vec{r}_0}$ are the empty sets, we obtain

$$\sum_{r_1} \mathrm{tr}_{I_{r_1}}[C_{Pr_1}] = 0, \tag{122}$$

which contradicts with the normalization condition in Eq. (112) of the QC-CC conditions. In the rest of the proof, we show Eq. (119) by induction with respect to m.

First, we show Eq. (119) for $m = M+N+1$ as follows. Since the operator $C_{P\vec{r}_{M+N}F}$ can be written as

$$C_{P\vec{r}_{M+N}F} = \sum_{i,j,k,l} |S\rangle\rangle^{PI_iO_iI'_kO'_kF} \langle\langle S|^{PI_jO_jI'_lO'_lF} \otimes C^{(ijkl)}_{P\vec{r}_{M+N}}, \tag{123}$$

where $C^{(ijkl)}_{P\vec{r}_{M+N}} := \sum_a |\tilde{\xi}^{(a),\vec{r}_{M+N}}_{ik}\rangle\!\rangle\langle\!\langle\tilde{\xi}^{(a),\vec{r}_{M+N}}_{jl}|$. The partial trace $\mathrm{tr}_F C_{P\vec{r}_{M+N}F}$ is given by

$$\begin{aligned}\mathrm{tr}_F[C_{P\vec{r}_{M+N}F}] = \sum_{i,j=1}^{M}\sum_{k,l=1}^{N} \Big(&|0\rangle\langle 0|^{P_C} \otimes |\mathbb{1}\rangle\!\rangle^{P_T I_i}\langle\!\langle\mathbb{1}|^{P_T I_j} \otimes |\mathbb{1}\rangle\!\rangle^{O_i I'_k}\langle\!\langle\mathbb{1}|^{O_j I'_l} \otimes \mathbb{1}^{O'_l\to O'_k} \\ &+ |1\rangle\langle 1|^{P_C} \otimes |\mathbb{1}\rangle\!\rangle^{P_T I'_k}\langle\!\langle\mathbb{1}|^{P_T I'_l} \otimes |\mathbb{1}\rangle\!\rangle^{O'_k I_i}\langle\!\langle\mathbb{1}|^{O'_l I_j} \otimes \mathbb{1}^{O_j\to O_i}\Big) \otimes C^{(ijkl)}_{P\vec{r}_{M+N}},\end{aligned} \tag{124}$$

i.e., Eq. (119) holds for $m = M+N+1$.

To complete the proof, we show Eq. (119) by assuming Eq. (119) for $m \leftarrow m+1$, i.e.,

$$\begin{aligned}\sum_{r_{m+1}} \mathrm{tr}_{I_{r_{m+1}}}[C_{P\vec{r}_m r_{m+1}}] = \sum_{i,j\in\mathbb{A}_{\vec{r}_m}}\sum_{k,l\in\mathbb{B}_{\vec{r}_m}} \Big(&|0\rangle\langle 0|^{P_C} \otimes |\mathbb{1}\rangle\!\rangle^{P_T I_i}\langle\!\langle\mathbb{1}|^{P_T I_j} \otimes |\mathbb{1}\rangle\!\rangle^{O_i I'_k}\langle\!\langle\mathbb{1}|^{O_j I'_l} \otimes \mathbb{1}^{O'_l\to O'_k} \\ &+ |1\rangle\langle 1|^{P_C} \otimes |\mathbb{1}\rangle\!\rangle^{P_T I'_k}\langle\!\langle\mathbb{1}|^{P_T I'_l} \otimes |\mathbb{1}\rangle\!\rangle^{O'_k I_i}\langle\!\langle\mathbb{1}|^{O'_l I_j} \otimes \mathbb{1}^{O_j\to O_i}\Big) \otimes C^{(ijkl)}_{P\vec{r}_m}.\end{aligned} \tag{125}$$

By symmetry with (I_i, O_i) and (I'_k, O'_k), it is sufficient to show if $r_m \in \{1,\ldots,M\}$ holds. From Eq. (111) [or Eq. (110) for $m = M+N$] in the QC-CC conditions and Eq. (125), we obtain

$$\begin{aligned}&\sum_{i,j\in\mathbb{A}_{\vec{r}_m}}\sum_{k,l\in\mathbb{B}_{\vec{r}_m}} \Big(|0\rangle\langle 0|^{P_C} \otimes |\mathbb{1}\rangle\!\rangle^{P_T I_i}\langle\!\langle\mathbb{1}|^{P_T I_j} \otimes |\mathbb{1}\rangle\!\rangle^{O_i I'_k}\langle\!\langle\mathbb{1}|^{O_j I'_l} \otimes \mathbb{1}^{O'_l\to O'_k} \\ &\qquad + |1\rangle\langle 1|^{P_C} \otimes |\mathbb{1}\rangle\!\rangle^{P_T I'_k}\langle\!\langle\mathbb{1}|^{P_T I'_l} \otimes |\mathbb{1}\rangle\!\rangle^{O'_k I_i}\langle\!\langle\mathbb{1}|^{O'_l I_j} \otimes \mathbb{1}^{O_j\to O_i}\Big) \otimes C^{(ijkl)}_{P\vec{r}_m} \\ &= \sum_{i,j\in\mathbb{A}_{\vec{r}_m}}\sum_{k,l\in\mathbb{B}_{\vec{r}_m}} |0\rangle\langle 0|^{P_C} \otimes |\mathbb{1}\rangle\!\rangle^{P_T I_i}\langle\!\langle\mathbb{1}|^{P_T I_j} \otimes \mathbb{1}^{O'_l\to O'_k} \otimes A_{ijkl} \\ &\qquad + |1\rangle\langle 1|^{P_C} \otimes |\mathbb{1}\rangle\!\rangle^{P_T I'_k}\langle\!\langle\mathbb{1}|^{P_T I'_l} \otimes |\mathbb{1}\rangle\!\rangle^{O'_k I_i}\langle\!\langle\mathbb{1}|^{O'_l I_j} \otimes B_{ijkl},\end{aligned} \tag{126}$$

where A_{ijkl} and B_{ijkl} are defined by

$$A_{ijkl} := \begin{cases} |\mathbb{1}\rangle\!\rangle^{O_i I'_k}\langle\!\langle\mathbb{1}|^{O_j I'_l} \otimes \tilde{C}^{(ijkl)}_{P\vec{r}_m} \otimes \mathbb{1}^{O_{r_m}} & (i,j \neq r_m) \\ \frac{1}{d} C^{(ijkl)}_{P\vec{r}_m} |\mathbb{1}\rangle\!\rangle^{I'_k O_{r_m}}\langle\!\langle\mathbb{1}|^{O_j I'_l} \otimes \mathbb{1}^{O_{r_m}} & (i = r_m \neq j) \\ \frac{1}{d} |\mathbb{1}\rangle\!\rangle^{I'_k O_i}\langle\!\langle\mathbb{1}|^{O_{r_m} I'_l} C^{(ijkl)}_{P\vec{r}_m} \otimes \mathbb{1}^{O_{r_m}} & (j = r_m \neq i) \\ \frac{1}{d} \mathbb{1}^{I'_l\to I'_k} \otimes C^{(ijkl)}_{P\vec{r}_m} \otimes \mathbb{1}^{O_{r_m}} & (i = j = r_m) \end{cases}, \tag{127}$$

$$B_{ijkl} := \begin{cases} \mathbb{1}^{O_j\to O_i} \otimes \tilde{C}^{(ijkl)}_{P\vec{r}_m} \otimes \mathbb{1}^{O_{r_m}} & (i,j \neq r_m) \\ \frac{1}{d} C^{(ijkl)}_{P\vec{r}_m} \mathbb{1}^{O_j\to O_{r_m}} \otimes \mathbb{1}^{O_{r_m}} & (i = r_m \neq j) \\ \frac{1}{d} \mathbb{1}^{O_{r_m}\to O_i} C^{(ijkl)}_{P\vec{r}_m} \otimes \mathbb{1}^{O_{r_m}} & (j = r_m \neq i) \\ C^{(ijkl)}_{P\vec{r}_m} \otimes \mathbb{1}^{O_{r_m}} & (i = j = r_m) \end{cases}, \tag{128}$$

$$\tilde{C}^{(ijkl)}_{P\vec{r}_m} := \frac{1}{d}\mathrm{tr}_{O_{r_m}} C^{(ijkl)}_{P\vec{r}_m}. \tag{129}$$

Using Lemma 6 for Eq. (126), we obtain

$$\sum_{k,l\in\mathbb{B}_{\vec{r}_m}} |\mathbb{1}\rangle\!\rangle^{O_i I'_k}\langle\!\langle\mathbb{1}|^{O_j I'_l} \otimes \mathbb{1}^{O'_l\to O'_k} \otimes C^{(ijkl)}_{P\vec{r}_m} = \sum_{k,l\in\mathbb{B}_{\vec{r}_m}} \mathbb{1}^{O'_l\to O'_k} \otimes A_{ijkl} \quad \forall i,j, \tag{130}$$

$$\mathbb{1}^{O_j\to O_i} \otimes C^{(ijkl)}_{P\vec{r}_m} = B_{ijkl} \quad \forall i,j,k,l. \tag{131}$$

From Eq. (131), we obtain

$$C^{(ijkl)}_{P\vec{r}_m} = \begin{cases} \tilde{C}^{(ijkl)}_{P\vec{r}_m} \otimes \mathbb{1}^{O_{r_m}} & (i,j \neq r_m) \\ 0 & (i = r_m \neq j \text{ or } j = r_m \neq i) \end{cases}, \tag{132}$$

where the cases of $i = r_m \neq j$ and $j = r_m \neq i$ are shown as below. If $i = r_m \neq j$ holds, from Eqs. (128) and (131), we obtain

$$\mathbb{1}^{O_j \to O_{r_m}} \otimes C^{(ijkl)}_{P\vec{r}_m} = \frac{1}{d} C^{(ijkl)}_{P\vec{r}_m} \mathbb{1}^{O_j \to O_{r_m}} \otimes \mathbb{1}^{O_{r_m}}. \tag{133}$$

By taking the inner product of Eq. (133) with $\mathbb{1}^{O_j \to O_{r_m}}$, we obtain

$$d C^{(ijkl)}_{P\vec{r}_m} = \frac{1}{d} C^{(ijkl)}_{P\vec{r}_m}, \tag{134}$$

i.e., $C^{(ijkl)}_{P\vec{r}_m} = 0$ holds for $i = r_m \neq j$. We can similarly show that $C^{(ijkl)}_{P\vec{r}_m} = 0$ for $j = r_m \neq i$. From Eq. (130) for $i = j = r_m$, we obtain

$$\sum_{k,l \in \mathbb{B}_{\vec{r}_m}} |\mathbb{1}\rangle\rangle^{O_{r_m} I'_k} \langle\langle \mathbb{1}|^{O_{r_m} I'_l} \otimes \mathbb{1}^{O'_l \to O'_k} \otimes C^{(ijkl)}_{P\vec{r}_m} = \sum_{k,l \in \mathbb{B}_{\vec{r}_m}} \mathbb{1}^{O'_l \to O'_k} \otimes \mathbb{1}^{I'_l \to I'_k} \otimes \frac{\mathbb{1}^{O_{r_m}}}{d} \otimes C^{(ijkl)}_{P\vec{r}_m} \quad \text{if } i = j = r_m. \tag{135}$$

Using Lemma 7, we obtain

$$C^{(ijkl)}_{P\vec{r}_m} = 0 \quad \text{if } i = j = r_m. \tag{136}$$

In conclusion, we obtain

$$C^{(ijkl)}_{P\vec{r}_m} = \begin{cases} \tilde{C}^{(ijkl)}_{P\vec{r}_m} \otimes \mathbb{1}^{O_{r_m}} & (i, j \neq r_m) \\ 0 & (\text{otherwise}) \end{cases}. \tag{137}$$

Thus, from Eqs. (111) and (125), we obtain

$$C_{P\vec{r}_m} = \sum_{i,j \in \mathbb{A}_{\vec{r}_{m-1}}} \sum_{k,l \in \mathbb{B}_{\vec{r}_{m-1}}} \Big(|0\rangle\langle 0|^{P_C} \otimes |\mathbb{1}\rangle\rangle^{P_T I_i} \langle\langle \mathbb{1}|^{P_T I_j} \otimes |\mathbb{1}\rangle\rangle^{O_i I'_k} \langle\langle \mathbb{1}|^{O_j I'_l} \otimes \mathbb{1}^{O'_l \to O'_k} \tag{138}$$

$$+ |1\rangle\langle 1|^{P_C} \otimes |\mathbb{1}\rangle\rangle^{P_T I'_k} \langle\langle \mathbb{1}|^{P_T I'_l} \otimes |\mathbb{1}\rangle\rangle^{O'_k I_i} \langle\langle \mathbb{1}|^{O'_l I_j} \otimes \mathbb{1}^{O_j \to O_i} \Big) \otimes \tilde{C}^{(ijkl)}_{P\vec{r}_m}. \tag{139}$$

Thus, defining $C^{(ijkl)}_{P\vec{r}_{m-1}}$ by

$$C^{(ijkl)}_{P\vec{r}_{m-1}} := \sum_{r_m} \mathrm{tr}_{I_{r_m}} [\tilde{C}^{(ijkl)}_{P\vec{r}_m}], \tag{140}$$

we obtain Eq. (119). We finish the proof by recalling that Eq. (119) implies Eq. (122), which contradicts the normalization condition in Eq. (112) of the QC-CC conditions. □

3.6 Lemma 6

Lemma 6. *The set of matrices*

$$\left\{ |\mathbb{1}\rangle\rangle^{P_T I'_k} \langle\langle \mathbb{1}|^{P_T I'_l} \otimes |\mathbb{1}\rangle\rangle^{O'_k I_i} \langle\langle \mathbb{1}|^{O'_l I_j} \otimes |\vec{\alpha}\rangle^{I'_{\bar{k}}} \left\langle \vec{\beta} \right|^{I'_{\bar{l}}} \otimes |\vec{\gamma}\rangle^{I_{\bar{i}}} \left\langle \vec{\delta} \right|^{I_{\bar{j}}} \right\}_{\substack{i,j \in \{1,\ldots,M\}, k,l \in \{1,\ldots,N\}, \\ \vec{\alpha},\vec{\beta} \in \{1,\ldots,d\}^{N-1}, \vec{\gamma},\vec{\delta} \in \{1,\ldots,d\}^{M-1}}} \tag{141}$$

is linearly independent if $\max(M, N) \leq d$ *holds. Similarly, the set of matrices*

$$\left\{ |\mathbb{1}\rangle\rangle^{P_T I_i} \langle\langle \mathbb{1}|^{P_T I_j} \otimes |\vec{\alpha}\rangle^{I_{\bar{i}}} \left\langle \vec{\beta} \right|^{I_{\bar{j}}} \right\}_{\substack{i,j \in \{1,\ldots,M\}, \\ \vec{\alpha},\vec{\beta} \in \{1,\ldots,d\}^{M-1}}} \tag{142}$$

is linearly independent if $M \leq d$ holds.

Proof. We consider the equation

$$\sum_{i,j,k,l,\vec{\alpha},\vec{\beta},\vec{\gamma},\vec{\delta}} A_{ijkl\vec{\alpha}\vec{\beta}\vec{\gamma}\vec{\delta}} |\mathbb{1}\rangle\rangle^{P_T I'_k} \langle\langle\mathbb{1}|^{P_T I'_l} \otimes |\mathbb{1}\rangle\rangle^{O'_k I_i} \langle\langle\mathbb{1}|^{O'_l I_j} \otimes |\vec{\alpha}\rangle^{I'_{\bar{k}}} \left\langle\vec{\beta}\right|^{I'_{\bar{l}}} \otimes |\vec{\gamma}\rangle^{I_{\bar{i}}} \left\langle\vec{\delta}\right|^{I_{\bar{j}}} = 0 \tag{143}$$

for complex coefficients $A_{ijkl\vec{\alpha}\vec{\beta}\vec{\gamma}\vec{\delta}}$. Since $\max(M, N) \leq d$ holds, for all $\vec{\alpha}, \vec{\beta}, \vec{\gamma}, \vec{\delta}$, there exists $\alpha^*, \beta^*, \gamma^*, \delta^* \in \{1, \ldots, d\}$ such that $\alpha^*, \beta^*, \gamma^*, \delta^*$ do not appear in $\vec{\alpha}, \vec{\beta}, \vec{\gamma}, \vec{\delta}$, respectively. By taking an inner product of Eq. (143) with $|\alpha^*\alpha^*\rangle^{P_T I'_k} \langle\beta^*\beta^*|^{P_T I'_l} \otimes |\gamma^*\gamma^*\rangle^{O'_k I_i} \langle\delta^*\delta^*|^{O'_l I_j} \otimes |\vec{\alpha}\rangle^{I'_{\bar{k}}} \left\langle\vec{\beta}\right|^{I'_{\bar{l}}} \otimes |\vec{\gamma}\rangle^{I_{\bar{i}}} \left\langle\vec{\delta}\right|^{I_{\bar{j}}}$ for any $i, j, k, l, \vec{\alpha}, \vec{\beta}, \vec{\gamma}, \vec{\delta}$, we obtain

$$A_{ijkl\vec{\alpha}\vec{\beta}\vec{\gamma}\vec{\delta}} = 0, \tag{144}$$

i.e., the set (141) is linearly independent. We can similarly show that the set (142) is linearly independent. □

3.7 Lemma 7

Lemma 7. *The set of matrices*

$$\left\{ \left(|\mathbb{1}\rangle\rangle^{O_{r_m} I'_k} \langle\langle\mathbb{1}|^{O_{r_m} I'_l} - \frac{\mathbb{1}^{O_{r_m}}}{d} \otimes \mathbb{1}^{I'_l \to I'_k} \right) \otimes \mathbb{1}^{O'_l \to O'_k} \otimes |\vec{\alpha}\rangle^{I'_{\bar{k}}} \left\langle\vec{\beta}\right|^{I'_{\bar{l}}} \otimes |\vec{\gamma}\rangle^{O'_{\bar{k}}} \left\langle\vec{\delta}\right|^{O'_{\bar{l}}} \right\}_{\substack{k,l \in \{1,\ldots,N\},\\ \vec{\alpha},\vec{\beta},\vec{\gamma},\vec{\delta} \in \{1,\ldots,d\}^{N-1}}} \tag{145}$$

is linearly independent if $N \leq \max(2, d-1)$ holds.

Proof. We numerically check the linear independence for the case $N = d = 2$ (see Listing 1). We prove the linear independence for the case $N \leq d - 1$ to complete the proof.

We consider the equation

$$\sum_{k,l,\vec{\alpha},\vec{\beta},\vec{\gamma},\vec{\delta}} A_{kl\vec{\alpha}\vec{\beta}\vec{\gamma}\vec{\delta}} \left(|\mathbb{1}\rangle\rangle^{O_{r_m} I'_k} \langle\langle\mathbb{1}|^{O_{r_m} I'_l} - \frac{\mathbb{1}^{O_{r_m}}}{d} \otimes \mathbb{1}^{I'_l \to I'_k} \right) \otimes \mathbb{1}^{O'_l \to O'_k} \otimes |\vec{\alpha}\rangle^{I'_{\bar{k}}} \left\langle\vec{\beta}\right|^{I'_{\bar{l}}} \otimes |\vec{\gamma}\rangle^{O'_{\bar{k}}} \left\langle\vec{\delta}\right|^{O'_{\bar{l}}} = 0 \tag{146}$$

for complex coefficients $A_{kl\vec{\alpha}\vec{\beta}\vec{\gamma}\vec{\delta}}$. Since $N \leq d - 1$ holds, for all $\vec{\alpha}, \vec{\beta}$, there exists $\alpha^*, \beta^* \in \{1, \ldots, d\}$ such that $\alpha^* \neq \beta^*$ holds and α^*, β^* do not appear in $\vec{\alpha}, \vec{\beta}$, respectively. By taking an inner product of Eq. (146) with $\frac{1}{d} |\alpha^*\alpha^*\rangle^{O_{r_m} I'_k} \langle\beta^*\beta^*|^{O_{r_m} I'_l} \otimes |\vec{\alpha}\rangle^{I'_{\bar{k}}} \left\langle\vec{\beta}\right|^{I'_{\bar{l}}} \otimes \mathbb{1}^{O'_l \to O'_k} \otimes |\vec{\gamma}\rangle^{O'_{\bar{k}}} \left\langle\vec{\delta}\right|^{O'_{\bar{l}}}$ for any $k, l, \vec{\alpha}, \vec{\beta}, \vec{\gamma}, \vec{\delta}$, we obtain

$$A_{kl\vec{\alpha}\vec{\beta}\vec{\gamma}\vec{\delta}} = 0, \tag{147}$$

i.e., the set (145) is linearly independent. □

Listing 1. MATLAB [11] code to check the linear independency of the set (145) for the case $d = 2$ and $N = 2$, which uses the functions from QETLAB [12].

```
clear

d=2;
N=2;

one = Tensor(IsotropicState(d, 1)*d,eye(d));
id = Tensor(eye(d)/d,eye(d),eye(d));
I = eye(d^(d-1));

for i = 1:d
    sys(i)=i;
```

```
end
PP = perms(sys);

pos=0;

% Calculate the set of matrices
for alpha = 1:d^(d-1)
    for beta=1:d^(d-1)
        for gamma = 1:d^(d-1)
            for delta=1:d^(d-1)
                for k = 1:size(PP,1)
                    for l=1:size(PP,1)
                        pos=pos+1;
                        A(:,:,pos) = Tensor(eye(d), PermutationOperator(d, PP(k,:)),
                            PermutationOperator(d, PP(k,:))) * PermuteSystems(Tensor(one-id, I(:,
                            alpha)*I(beta,:), I(:,gamma)*I(delta,:)), [1 2 4 3 5]) * Tensor(eye(
                            d), PermutationOperator(d, PP(l,:)), PermutationOperator(d, PP(l,:)));
                    end
                end
            end
        end
    end
end

% Flatten the matrices to vectors
for pos = 1:size(A,3)
    B(:,pos) = reshape(A(:,:,pos), [], 1);
end

rank(B) == size(B,2)
```

Supplementary Note 4 – Possible restricted simulations for qubit channels

From this section onwards, we revert to our original notation.

Using numerical methods, we have found three particular cases where a restricted simulation of the quantum switch acting on qubit channels *is possible*, exactly and deterministically, using a quantum comb. These are the cases of:

- Four identical calls to general qubit channels, called the order AAAA.
- Two calls to unitary qubit channels A and two calls to unitary qubit channels B, in the order AABB.
- Three calls to unitary qubit channels A and one call to unitary qubit channels B, in the order BAAA.

All of these results were obtained by numerically evaluating the equivalent of the primal SDP of the main text [Eq. (16)] in the case where the input states are fixed and the output target system is discarded, and finding that $p = 1$ up to a high numerical precision.

Effectively, this scenario amounts to an SDP that is analogous to the primal SDP in Eq. (16) of the main text, but with the first constraint written as

$$C_s * \left[(J_i^A)^{\otimes k_A} \otimes (J_j^B)^{\otimes k_B}\right] = p\,\mathrm{tr}_{t_O}(S_{+0}) * (J_i^A \otimes J_j^B) \quad \forall\, i, j, \tag{148}$$

where S_{+0} is defined as in Eq. (3). In this case, we have that $C_s, C \in \mathcal{L}(\mathcal{H}^{A_I} \otimes \mathcal{H}^{A_O} \otimes \mathcal{H}^{B_I} \otimes \mathcal{H}^{B_O} \otimes \mathcal{H}^{c_O})$ and $d_{c_I} = d_{t_I} = 1$.

In the case of 4 identical copies of general qubit channels, the input channels were given as a basis constructed in the form of Eq. (22) of the main text. The dimension of the space spanned by $k = 4$ identical copies of a general qubit channel is $d_{\mathcal{I}} = 1820$, a value that can be obtained from the expression presented in the Methods section of the main text.

In the case of k identical copies of qubit unitary channels, a basis can be constructed numerically by randomly sampling a set of qubit unitaries according to the Haar measure, guaranteeing that they are linearly independent. It is only necessary to know the dimension of this subspace beforehand to determine how many unitaries must be sampled.

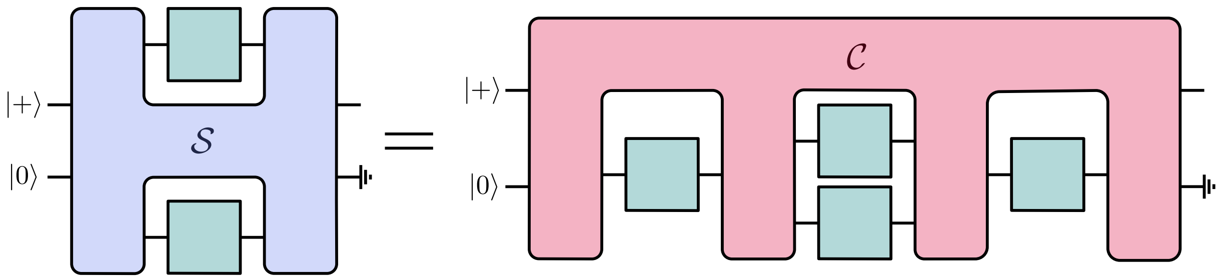

Supplementary Figure 5. **Semi-parallelized strategy for restricted qubit simulations.** The quantum switch acting on qubit channels can be deterministically simulated by a 4-slot quantum comb that has its second and third slots parallelized in a restricted scenario where the input control and target systems are fixed and the output target system is discarded in three different cases: for identical general quantum channels (i.e. when $A = B$) called order AAAA, and for unitary channels (i.e. when $A = U_A$ and $B = U_B$) in the orders AABB and BAAA.

The dimension of the linear space spanned by k copies of d-dimension unitary channels is the quantity $D(d, r, s)$ of Ref. [13] for the case $r = s = k$. For $d = 2$, the dimension of the subspace spanned by k identical copies of a unitary channel is given by $d_{\mathcal{U}} = D(d = 2, k, k) = \binom{2k+3}{3}$, which implies $d_{\mathcal{U}} = 10$ for $k = 1$, $d_{\mathcal{U}} = 35$ for $k = 2$, and $d_{\mathcal{U}} = 84$ for $k = 3$.

By numerically evaluating the appropriate SDP, we obtain that the maximum probability of success is $p = 1$ with very high precision in all aforementioned cases. The numerical precision was evaluated in the following way: In all three cases, all inequality constrains are strictly satisfied; as for the equality constraints, they are satisfied up to an error of at most 10^{-9} in the operators norm for the case of identical general qubit channels AAAA, and at most 10^{-7} in the cases of qubit unitary channels AABB and BAAA.

We also found that the maximum probability of success remains $p = 1$, with the same precision, in all three cases when the second and third slots of the quantum comb $\mathcal{C}$ are parallelized, as depicted in Fig. 5.

Impossible partly restricted simulation. As mentioned in the main text, we also found that when the output target system is not discarded—i.e., the partly restricted simulation scenario—then a deterministic simulation is no longer possible in all 3 aforementioned cases.

More concretely, this is the scenario where the first constraint in the primal SDP of the main text [Eq. (16)] is written as

$$C_s * \left[(J_i^A)^{\otimes k_A} \otimes (J_j^B)^{\otimes k_B}\right] = p\, S_{+0} * (J_i^A \otimes J_j^B) \quad \forall\, i, j, \tag{149}$$

where S_{+0} is defined as in Eq. (3), $C_s, C \in \mathcal{L}(\mathcal{H}^{A_I} \otimes \mathcal{H}^{A_O} \otimes \mathcal{H}^{B_I} \otimes \mathcal{H}^{B_O} \otimes \mathcal{H}^{t_O} \otimes \mathcal{H}^{c_O})$, and $d_{c_I} = d_{t_I} = 1$.

Evaluating the above SDP, for the partly restricted scenario, we found that $p < 1$ in the AAAA case of identical general qubit channels, the AABB case of qubit unitary channels, as well as the BAAA case of qubit unitary channels. Due to limitations in computational power, the partly restricted simulation SDP was evaluated in all three cases with an input that corresponds to a subset of the basis that was given as input to the restricted simulation SDP. Therefore, the solutions for the maximal probability of success correspond to upper bounds, and are given by

$$\text{order} = \text{AAAA (qubit, general)}, \quad p \lesssim 0.942 \tag{150}$$

$$\text{order} = \text{AABB (qubit, unitary)}, \quad p \lesssim 0.822 \tag{151}$$

$$\text{order} = \text{BAAA (qubit, unitary)}, \quad p \lesssim 0.667. \tag{152}$$

The difference between the two simulation scenarios discussed in this section is whether or not the output target state is discarded. Here we can see that this distinction in the requirements of the simulation is sufficient to transform cases where a deterministic simulation is possible (with a discarded output target system) into cases where a deterministic simulation is no longer possible (without discarding the output target system).

Supplementary Note 5 – No-go results for unitary channels

As previously discussed, the simulation of the action of the quantum switch on unitary channels is possible when an extra call of one of the input channels is available. This was first shown by Ref. [9] for the action of the quantum switch on entire single-party unitary channels, and here we have shown this result to extend to a more general scenario where the quantum switch acts only on part of the input bipartite unitary channels (see Theorem 1 in Sec. 2 2.4). These results hold in the general simulation scenario, where the input systems are not fixed and the output systems are not discarded, and hold as well for any dimension.

However, this result crucially depends not only on the number of extra calls available, but also on the order in which the quantum unitary channels are applied in the simulation. The cases where a simulation is possible for either single-party or for bipartite unitary channels with $(k_A, k_B) = (2, 1)$ use the order ABA. If the order of the applied channels is instead either AAB or BAA, we find that a deterministic simulation is no longer possible, even in the restricted qubit scenario.

Moreover, the existence of a simulation for unitary channels in the order ABA implies that, when $(k_A, k_B) = (2, 2)$, a simulation with the orders ABAB and ABBA also trivially exists. In Sec. 4, we showed that a simulation using the order AABB also exists for unitary channels, albeit *only* in the restricted qubit case where the output target system is discarded. For the case where $(k_A, k_B) = (3, 1)$, a simulation in the orders AABA and ABAA exists, following trivially from the result for ABA. We showed in Sec. 4 that a simulation for unitary channels in the order BAAA exists as well, but once again *only* in the restricted qubit case. The one case left to study then is a simulation of the quantum switch acting on unitary channels in the order AAAB. We show such a simulation is not possible.

Hence, our three no-go results for simulations of the quantum switch acting exclusively on unitary channels concern the cases of:

- Two calls of the unitary channels A and one call of unitary channels B, in the order AAB.
- Two calls of the unitary channels A and one call of unitary channels B, in the order BAA.
- Three calls of the unitary channels A and one call of unitary channels B, in the order AAAB.

In order to show these results, we numerically evaluate the maximum probability of success of simulating the quantum switch when the input channels are unitary. We do so in the case where the input channels are acting on qubit systems, i.e., when $d = 2$, and in the restricted simulation scenario where the input control and target systems are fixed, and the output target system is discarded [see Eq. (148)]. We remark once again that the impossibility of a deterministic simulation in the restricted, fixed-dimension case implies the impossibility of a deterministic simulation in general.

In this case, we do not construct an explicit basis analytically for the subspace spanned by k identical copies of a qubit unitary channel, but instead, we randomly sample a set of $d_{\mathcal{I}}$ linearly independent qubit unitary channels $\{U_i^{\otimes k}\}_{i=1}^{d_{\mathcal{I}}}$ to form our basis. We repeat here that for the case where $k = 1$, $d_{\mathcal{U}} = 10$, for $k = 2$, $d_{\mathcal{U}} = 35$, and for $k = 3$, and $d_{\mathcal{U}} = 84$. This implies that in the $(k_A, k_B) = (2, 1)$ case, one needs $35 \cdot 10 = 350$ pairs of qubit unitary channels and for $(k_A, k_B) = (3, 1)$, one needs $84 \cdot 10 = 840$ pairs of qubit unitary channels as input for the primal and dual SDPs [Eqs. (16) and (17) of the main text]. We numerically obtain the values of

$$\text{order} = \text{AAB} \quad \text{(qubit, unitary)}, \quad p \approx 0.600 \tag{153}$$

$$\text{order} = \text{BAA} \quad \text{(qubit, unitary)}, \quad p \approx 0.851 \tag{154}$$

$$\text{order} = \text{AAAB} \quad \text{(qubit, unitary)}, \quad p \approx 0.708. \tag{155}$$

Supplementary Note 6 – Efficient certification that a matrix is positive semidefinite

A self-adjoint linear operator $A \in \mathcal{L}(\mathbb{C}^d)$ is positive semidefinite if and only if there exists an operator $L \in \mathcal{L}(\mathbb{C}^d)$ such that $A = LL^\dagger$. Moreover, the operator L can be taken to be a lower triangular matrix, i.e., a matrix in which all entries above the main diagonal are zero. The decomposition of a positive semidefinite matrix as $A = LL^\dagger$ is referred to as the Cholesky decomposition, and finding such a decomposition can be done efficiently [14].

In the final step of the algorithm used for computer-assisted proofs presented in the Methods section of the main text, one is required to certify that an operator A is positive semidefinite. When A is stored as a symbolic matrix, due

to the way computers manipulate symbolic variables, if the matrix A is not sparse enough, ensuring that $A \geq 0$ may be a prohibitively time-consuming task even when using the Cholesky decomposition. This was the case, for instance, when trying to ensure that the matrix $\overline{\mathbb{P}}(\Gamma^{\mathtt{sym}}) + \eta\mathbb{1} - \sum_{i,j} R_{ij}^{\mathtt{OK}} \otimes (J_i^{A^{\otimes k_A}} \otimes J_j^{B^{\otimes k_B}})^T$ in our computer-assisted proofs algorithm involving $k_A + k_B = 4$-slot quantum combs. Below, we describe an algorithm that can be used to ensure that a symbolic matrix A is positive semidefinite, which is considerably faster than performing Cholesky decomposition on a symbolic matrix A.

The algorithm we present below is based on three key ideas.

1. It is possible to rigorously certify that a matrix is positive definite using floating-point arithmetic quickly. One way to attain this goal is to use the methods presented in Ref. [15]. It shows how to efficiently certify that a matrix A is positive definite using a rigorous algorithm that accounts for all possible computational and rounding errors and remains valid in the presence of underflow.

2. If a matrix A is "close" to another matrix A', and A' is "far" from the set of non-positive semidefinite matrices, then A has to be positive semidefinite.

3. If A is matrix with symbolic entries, we can obtain a floating-point variable matrix A' that is guaranteed to be close to A. This can be done via arbitrary-precision arithmetic [16].

<u>Algorithm to prove that a symbolic self-adjoint matrix A is positive semidefinite:</u>

1. `Construct a self-adjoint matrix` A' `that is equal to` A `up to` n `decimal digits`
   This step can be accomplished using arbitrary-precision arithmetic [16] with a precision of n decimal digits. In this way, all matrix elements of $A' - A$ are between -10^{-n+1} and 10^{-n+1}, ensuring that

$$-J \cdot 10^{-n+1} \;\leq\; A' - A \;\leq\; J \cdot 10^{-n+1}, \tag{156}$$

   where $J \geq 0$ is a matrix in which all entries are the number one.

2. `Prove that` $A' - J \geq 0$ `using the algorithm presented in Ref. [15].`
   Since $A' - J \cdot 10^{-n+1} \leq A$ holds, if we ensure that $A' - J \geq 0$, then by transitivity it follows that $A \geq 0$.

[1] M. Araújo, C. Branciard, F. Costa, A. Feix, C. Giarmatzi, and Č. Brukner, Witnessing causal nonseparability, New J. Phys. **17**, 102001 (2015), arXiv:1506.03776 [quant-ph].
[2] S. Milz and M. T. Quintino, Characterising transformations between quantum objects, 'completeness' of quantum properties, and transformations without a fixed causal order, Quantum **8**, 1415 (2024), arXiv:2305.01247 [quant-ph].
[3] J. Wechs, H. Dourdent, A. A. Abbott, and C. Branciard, Quantum Circuits with Classical Versus Quantum Control of Causal Order, PRX Quantum **2**, 030335 (2021), arXiv:2101.08796 [quant-ph].
[4] M. Ozawa, Quantum measuring processes of continuous observables, J. Math. Phys. **25**, 79 (1984).
[5] M. M. Wilde, *Quantum Information Theory*, 2nd ed. (Cambridge University Press, 2017) arXiv:1106.1445 [quant-ph].
[6] F. Buscemi, K. Kobayashi, S. Minagawa, P. Perinotti, and A. Tosini, Unifying different notions of quantum incompatibility into a strict hierarchy of resource theories of communication, Quantum **7**, 1035 (2023), arXiv:2211.09226 [quant-ph].
[7] J. Watrous, *The Theory of Quantum Information* (Cambridge University Press, 2018).
[8] S. Yoshida, A. Soeda, and M. Murao, Reversing Unknown Qubit-Unitary Operation, Deterministically and Exactly, Phys. Rev. Lett. **131**, 120602 (2023), arXiv:2209.02907 [quant-ph].
[9] G. Chiribella, G. M. D'Ariano, P. Perinotti, and B. Valiron, Quantum computations without definite causal structure, Phys. Rev. A **88**, 022318 (2013), see also version arXiv:0912.0195v1 [quant-ph].
[10] W. Yokojima, M. T. Quintino, A. Soeda, and M. Murao, Consequences of preserving reversibility in quantum superchannels, Quantum **5**, 441 (2021), arXiv:2003.05682 [quant-ph].
[11] The MathWorks Inc., MATLAB version: 9.13.0 (R2022b) (2022).
[12] N. Johnston, QETLAB: A MATLAB toolbox for quantum entanglement, version 0.9, `https://qetlab.com` (2016).
[13] A. Roy and A. J. Scott, Unitary designs and codes, Des. Codes Cryptogr. **53**, 13–31 (2009), arXiv:0809.3813 [math.CO].
[14] https://en.wikipedia.org/wiki/cholesky_decomposition, accessed on 26/09/2024.
[15] S. M. Rump, Verification of positive definiteness, BIT Numer. Math. **46**, 433 (2006).
[16] https://en.wikipedia.org/wiki/arbitrary-precision_arithmetic, accessed on 26/09/2024.
